# Machine Learning-Driven Discovery of Key Descriptors for CO_2_ Activation over Two-Dimensional Transition Metal Carbides and Nitrides

**DOI:** 10.1021/acsami.3c02821

**Published:** 2023-06-19

**Authors:** B. Moses Abraham, Oriol Piqué, Mohd Aamir Khan, Francesc Viñes, Francesc Illas, Jayant K. Singh

**Affiliations:** † Department of Chemical Engineering, Indian Institute of Technology Kanpur, Kanpur 208016, India; ‡ Departament de Ciència de Materials i Química Física, Institut de Química Teòrica i Computacional (IQTCUB), Universitat de Barcelona, c/ Martí i Franquès 1-11, Barcelona 08028, Spain; § Prescience Insilico Private Limited, Bangalore 560049, India

**Keywords:** MXenes, CO_2_ activation, machine learning, density functional calculations, descriptors

## Abstract

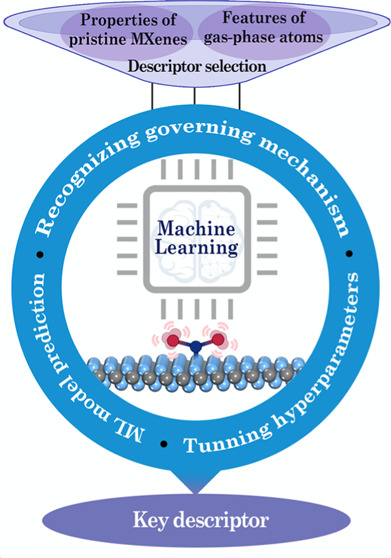

Fusing high-throughput quantum mechanical screening techniques with modern artificial intelligence strategies is among the most fundamental —yet revolutionary— science activities, capable of opening new horizons in catalyst discovery. Here, we apply this strategy to the process of finding appropriate key descriptors for CO_2_ activation over two-dimensional transition metal (TM) carbides/nitrides (MXenes). Various machine learning (ML) models are developed to screen over 114 pure and defective MXenes, where the random forest regressor (RFR) ML scheme exhibits the best predictive performance for the CO_2_ adsorption energy, with a mean absolute error ± standard deviation of 0.16 ± 0.01 and 0.42 ± 0.06 eV for training and test data sets, respectively. Feature importance analysis revealed *d*-band center (ε_d_), surface metal electronegativity (χ_M_), and valence electron number of metal atoms (*M*_V_) as key descriptors for CO_2_ activation. These findings furnish a fundamental basis for designing novel MXene-based catalysts through the prediction of potential indicators for CO_2_ activation and their posterior usage.

## 1 Introduction

The excessive carbon dioxide (CO_2_) concentration in Earth’s atmosphere has become a large threat to the environment given its main role in global warming; therefore, a lot of efforts have been taken worldwide to remove it. The rise of CO_2_ concentration in the atmosphere is mainly due to the massive destruction of forests as well as the extensive exploitation of fossil fuels, which led to a continuous increase of CO_2_ concentration, that will reach ∼590 ppm by the year 2100, causing an expected global temperature raise by 1.9 °C, with the concomitant acidification of oceans and devastating consequences for the marine ecosystems.^1^ At present, the increasing CO_2_ emissions are partly controlled through either converting it into useful carbon-based fuels/chemicals or by storing it in a stabilized media. To attain a valid impact on both environment and economy, it is necessary to utilize CO_2_ instead of just storing it, to thus unlock its potential and trigger profitable industrial applications. Hitherto, several types of catalysts were investigated aimed at CO_2_ activation and reduction, including different metal oxides, pure metals and alloys, organometallics, single-atom catalysts, non-metals, and nano-metals.^2−4^ Typically, metals such as Cd, Sn, In, Pd, and Bi mediate the formation of formic acid from CO_2_,^5−8^ while Ti, Zn, and Au can efficiently convert CO_2_ into CO.^9−11^ These studies demonstrated that the CO_2_ molecule can interact with metal surfaces through either strong or weak binding modes. In the case of strong interactions, the metal–carbon (M–C) overbonding may poison the catalyst surface, making the active sites inaccessible for further reduction of CO_2_ with a concomitant reduction of product formation. In contrast, a weak bonding between CO_2_ and a given metal surface does not allow for the CO_2_ C–O bond dissociation, as desorption prevails over this bond scission chemical step, which would favor the formation of the desired products. It should be borne in mind that the C–O bond enthalpy in the CO_2_ molecule is very large, of 803 kJ·mol^–1^,^12^ and thus the activation of CO_2_ can be regarded as a suitable approach to lower the CO_2_ reaction conditions and energy demands. Therefore, a thorough activation analysis is highly required when designing novel catalysts based on rational approaches, to uncover which factors govern both activity and selectivity during the reactive processes.

In general, the CO_2_ binding energy over a potential catalytic surface is considered as an effective source for predicting the likelihood of CO_2_ reduction reactions.^13^ However, the experimental accurate measurement of the CO_2_ binding energy is far from being a simple issue.^14^ On the other hand, the theoretical modeling of the catalytic activity on a given material surface requires extensive yet accurate calculations, preferably from first principles-based methods, leading to a good understanding of the interaction of CO_2_ with the surface of interest and accounting also for coverage effects, but at a high computational cost, though. In this regard, properties that provide information about the catalytic activity from a lower computational cost are highly preferable, particularly to screen over a pool of chemically related family of materials. In a simple case scenario, the adsorption energies can be linearly correlated with electronic descriptors that only require investigating the substrates, *e.g.*, by density functional theory (DFT), significantly reducing the computation cost for predicting the catalytic activity. In particular, Hammer and Nørskov^15^ proposed one of the most successful descriptors to date, the *d*-band center, capable of predicting the adsorption energy of a given adsorbate at different TM surfaces using information of the TM surface electronic structure only. Here, the essence of the statement indicates that the binding energy of CO_2_ to the TM surface does not require entire details of the density of sates, where instead the *d*-band center is sufficient to correlate the interaction strength with the surface chemical activity and, eventually, the catalytic performance. In addition, other structural parameters such as bond lengths and angles, even surface coordination numbers, could be correlated with adsorption energies. By mapping the adsorption energy with materials intrinsic properties, one can obtain descriptors that do not only provide a fast screening over them with a rather high accuracy but also offer fundamental insights into the coupling between CO_2_ and the surfaces of interest.

In the recent years, machine learning (ML) models trained on a limited number of quantum-mechanical calculations have become an appealing alternative for high-throughput prediction of chemical reactivity with either algorithm-derived^16−20^ or handcrafted features.^21−23^ The input variables required for ML modeling are typically accessible from the relaxed pristine materials surface structures without the presence of adsorbates. Using such properties with a low computational cost, one can predict complex parameters such as catalytic activities or adsorption energy distributions in a much faster way. Since the ML analysis within the catalysis field mainly deals with particular chemical or physical properties, *e.g*., adsorption energies, *d*-band centers, selectivities, limiting potentials, and so on, it is essential to consider supervised ML algorithms that map the target data set. Linear regression is a simple procedure with a highly potential and widespread approach used to analyze descriptors and to establish scaling relations for predicting valuable information in the computational heterogeneous catalysis field. More advanced techniques are currently available to handle multiple features such as non-linear relationships,^24−27^ including kernel ridge regression,^28^ neural networks,^29^ random forest regression,^30^ and Gaussian processes regression,^31^ to name a few. Ultimately, choosing suitable descriptors is essential in any ML to regulate the prediction power and the learning efficiency.^32^

In this work, ML models are developed to mine and map the CO_2_ activation over pure and defective MXenes based solely on their pristine properties and the features of gas-phase atoms that enter in the MXene chemical composition. Note in passing by that for CO_2_ activation, we refer here to a strong interaction between the CO_2_ molecule and the MXene surfaces, leading to significant changes in the adsorbed CO_2_ geometry, including a bent geometry with elongated C–O bonds and a molecular negative charge, resulting from a charge transfer from the MXene surface to CO_2_. This CO_2_ activation must not be misled with another widely used meaning, implying the CO_2_ conversion into other chemicals, *e.g.*, CO, formic acid, methanol, and so on, although both definitions are connected, since the bent CO_2_ geometry is quite often the key, decisive state in CO_2_ conversion, as found in organometallics,^33^ TM carbides,^34^ MXenes,^35^ metals,^36^ alloys,^37^ and oxide-based catalysts.^38,39^

**Figure 1:**
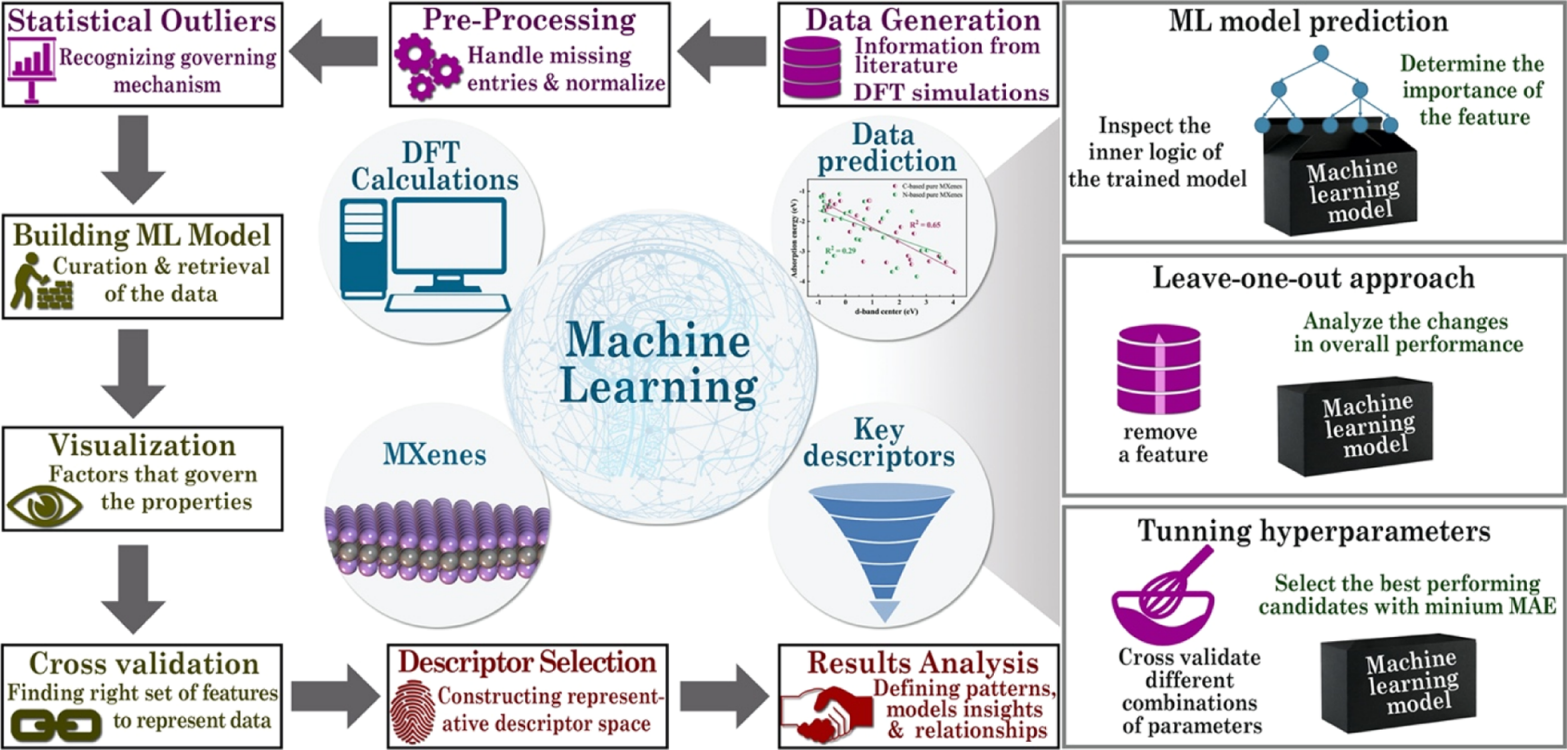
Schematic diagram of the ML workflow, trained on a data set generated from previous work and DFT calculations, aimed at identifying potential descriptors for CO_2_ activation over two-dimensional TM carbides and nitrides (MXenes).

Thus, the fundamental goal of the present study is to develop and understand ML models for activated CO_2_ adsorption on MXenes, which can be quantitatively implemented and leveraged for the predictive analysis in drawing useful information into the process of CO_2_ posterior conversion. Figure 1 displays the schematic diagram of the ML workflow, trained on a data set generated from our previous literature and DFT calculations to identify potential descriptors for CO_2_ activation over MXenes. To this end, three regression models, namely, multivariate linear regression (MLR), decision tree regression (DTR), and random forest regression (RFR) are set up and evaluated with the aim of predicting potential descriptors for CO_2_ activation over these materials.^40^ Accordingly, we performed a feature importance evolution and investigated the effect of each primary feature on the target adsorption properties. As demonstrated below, the RFR model is best performing, using *d*-band center, ε_d_, the MXene surface metal electronegativity, χ_M_, and valence electron number of metal atoms, *M*_V_, as meaningful features to predict the activation of CO_2_ for the chosen MXene class of materials. This high-throughput screening research based on first-principles calculations and ML predictions can discover prominent indicators of CO_2_ activation over MXene materials, and it is likely to be transferred to other bulk TM carbides/nitrides materials as well.

## 2 Methodology

### 2.1 Data Collection and Pre-processing

**Figure 2:**
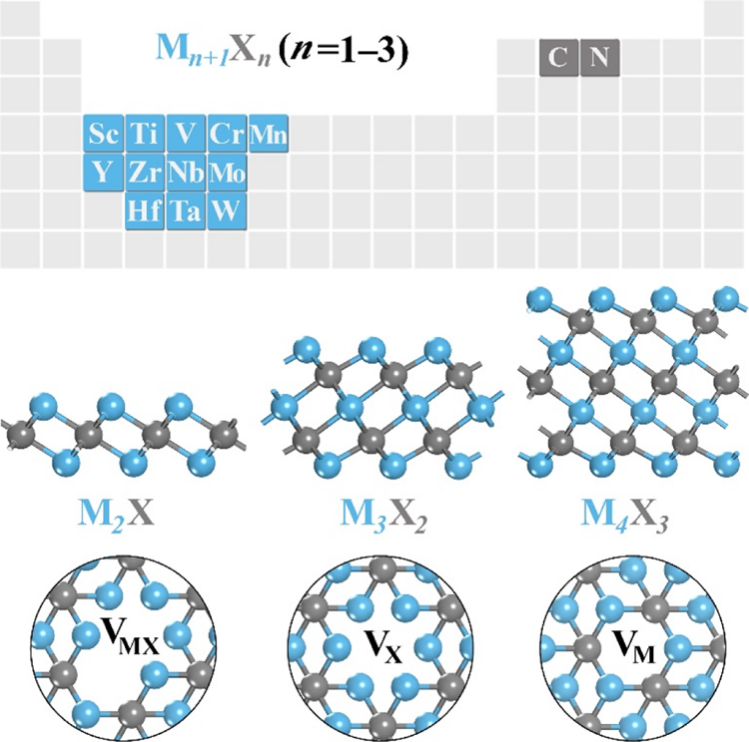
Side and top atomic structure views of TM carbides and nitrides MXenes with formula M_*n*+1_X_*n*_ (*n* = 1–3) with X = C or N and M metals from groups III–V of the Periodic Table. A total of 114 data points are extracted, among which 60 points are from pure MXenes with varying thickness, while remaining points correspond to MXenes with different vacancies; metal vacancy (*V*_M_), carbon/nitrogen vacancy (*V*_X_), and metal and nearby carbon/nitrogen vacancy (*V*_MX_).

The data required to nurture the developed ML tools were collected from our previous literature on MXenes for CO_2_ capture.^35,41−44^ A total of 114 data points were extracted, among which 60 points are from pure MXenes with varying thickness, while remaining 54 points correspond to MXenes with different sorts of vacancies; see Figure 2. Note that MXenes are usually surface-terminated as a result of the synthesis procedure, yet bare MXenes are nowadays attainable either through molten salts synthesis^45^ or after applying cleaning protocols.^46^ Furthermore, such non-terminated sites have been appointed to be key catalytic active centers in CO_2_ conversion, as shown in the dry methane reforming.^47^ In addition, some previous cases, where *CO_2_ was found to dissociate into *CO and *O adsorbates upon relaxation on the MXene surfaces —due to a molecular placement too close to the MXene surface, and so a higher energy level, which led the dissociation— were reoptimized in order to gain a stable *CO_2_ adsorption state. In addition, we also observed that a substantial amount of data was missing, particularly on surface descriptors, which were here calculated and completed; see below. Notice that the data source had many aspects in common, *e.g.*, all being DFT calculations on *p*(3×3) slab models, with a minimum vacuum of 10 Å, and using Perdew–Burke–Ernzerhof (PBE) exchange–correlation functional,^48^ with Grimme’s D3 correction to account for dispersive forces.^49^ However, data slightly differed concerning the plane-wave basis set kinetic energy-cutoff or the Brillouin zone **k**-points density. To assess the possible effect of such input differences on binding energies, we carried out test evaluations on 11% of the data set using the same materials, with representatives from pure MXenes and varying thicknesses and cases including different sorts of vacancies. The evaluated impact on target properties such as adsorption energies, bond lengths, and O=C=O angles were found to be at most of 0.07 eV, 0.03 Å, and 5.41°, respectively. Such discrepancies are well below or at least comparable to the inherent DFT accuracy.

The entire set of data points was then split into randomly selected training and test subsets. Accordingly, a random 20% of the total data points were labeled as test data and the remaining 80% was labeled as training data for the evaluation of the designed models. To better understand the importance of the studied models with the set of primary features, we considered the Pearson correlation coefficient, *R*, and the mean absolute error (MAE) as main evaluation indices.

### 2.2 ML Models and Hyperparameter Tuning

Three ML models, namely, MLR, DTR, and RFR, were devised and evaluated to predict the CO_2_ activation over MXenes on the set of described input features or descriptors. Based on a training data set, each model was developed, where the test data set was employed to evaluate their prediction accuracy. For more detailed information about the considered three ML models; see Section S1 of the Supporting Information. To improve the model prediction quality, a cross-validation was carried out during the training process to tune the hyperparameters. Generally, the hyperparameter tuning (HT) is used for obtaining optimal model performance by finding a set of hyperparameters, which are tuned during the model training process,^50^*e.g.*, the DTR and RFR branches and leaf nodes; see Figures S1 and S2 in the Supporting Information. In the present study, HT was carried out using a grid search method, which is reliable methodology, while tuning a lower set of primary features. All the data processing and ML technique implementation were performed using the open-source scikit-learn library.^51^

### 2.3 DFT Calculations

Complementary periodic DFT calculations were carried out using the Vienna *ab initio* simulation package (VASP) code,^52^ using a plane wave basis set for the valence electron density with an optimal kinetic energy cutoff of 415 eV. For the scalar-relativistic treatment of the effect of core electrons on the valence density, projector augmented wave^53^ pseudopotentials were used. A generalized gradient approximation exchange–correlation functional has been employed, in particular, that proposed by Perdew-Burke-Ernzerhof (PBE).^48^ The geometry optimization was considered converged when forces acting on atoms were all below 0.01 eV·Å^–1^, while an electronic convergence criterion of 10^–5^ eV was imposed. An optimal Monkhorst–Pack grid of **k**-points of 5×5×1 dimensions was used, overall guaranteeing adsorption energies to be converged below chemical accuracy of 1 kcal·mol^–1^, *ca.* 0.04 eV. Dispersive forces were accounted using Grimme’s D3 method,^49^ being PBE-D3 a suited level of calculation employed in previous studies.^41−44^

The adsorption energy, *E*_ads_, of CO_2_ on various MXene surfaces was obtained from the following equation
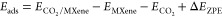
1where *E*_CO_2_/MXene_, *E*_MXene_, and *E*_CO_2__ are the total energies of CO_2_ adsorbed on the corresponding MXene surface, that of the relaxed pristine MXene, and that of the isolated CO_2_ molecule, respectively. For the CO_2_ molecule, it was placed within a symmetric box of 10×10×10 Å dimensions and optimized at the **Γ**-point. Δ*E*_ZPE_ is the zero point energy (ZPE) difference in between the adsorbed CO_2_ and that of the gas phase within the harmonic approximation. For further details, we refer to literature.^35^

As far as descriptors are concerned, the work function, ϕ, is defined as the amount of energy required to move an electron from the material Fermi level, *E*_F_, and place it in the vacuum energy level, *E*_vac_. Thus
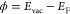
2

In the *d*-band center model, it is defined as the gravimetric center of the d-projected density of states of a surface TM atom, within the initial energy level up to the energy level corresponding to an hypothetical *d*^10^ electronic configuration of the TM; see further details in literature.^54^ Aside, a Bader’s atoms-in-molecules electronic density analysis is carried out to integrate it within regions whose charge is assigned to certain atoms.^55^ Thus, a negative *Q* value implies a negative charge, and *vice versa*. Finally, the exfoliation energies, *E*_exf_, are gained, computed as the energy necessary to remove the A element from MXene MAX phase precursors,^44^ and obtained as follows
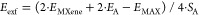
3where *E*_MXene_ and *E*_MAX_ are the isolated MXene and the MAX unit cell total energies, respectively, as depicted in Figure S3 of the Supporting Information. Besides, *E*_A_ and *S*_A_ indicate the bulk phase atomic energy of A species and the cross-section area of each created MXene unit, respectively. Within this definition, the larger the *E*_exf_, the stronger the bonding between MXene layers and the A phase and the costlier is to separate them.

## 3 Results and Discussion

Having consistently gained and gathered all the necessary data, we first considered four target variable indicators of the CO_2_ activation. These included CO_2_ adsorption energy, *E*_ads_, in the sense that, *a priori*, the stronger the bonding, the higher the activation. Aside from this energetic feature, we regarded two geometric parameters, the average C–O bond distance, *d*(CO), and the CO_2_ molecular angle, α(OCO), since, ideally, the activated CO_2_ features a reduced angle compared to the linear gas molecule angle of 180°, plus elongated C–O bonds, result from the activated bent geometry, and a consequence of a charge transfer from the substrate material.^56,57^ Thus, the smaller the angle and the larger the bond lengths, the more activated the CO_2_. Finally, the mentioned charge transfer can be quantified through the Bader charge of the adsorbed CO_2_, *Q*, in the sense that, the larger the charge, the more activated CO_2_ is.

**Figure 3:**
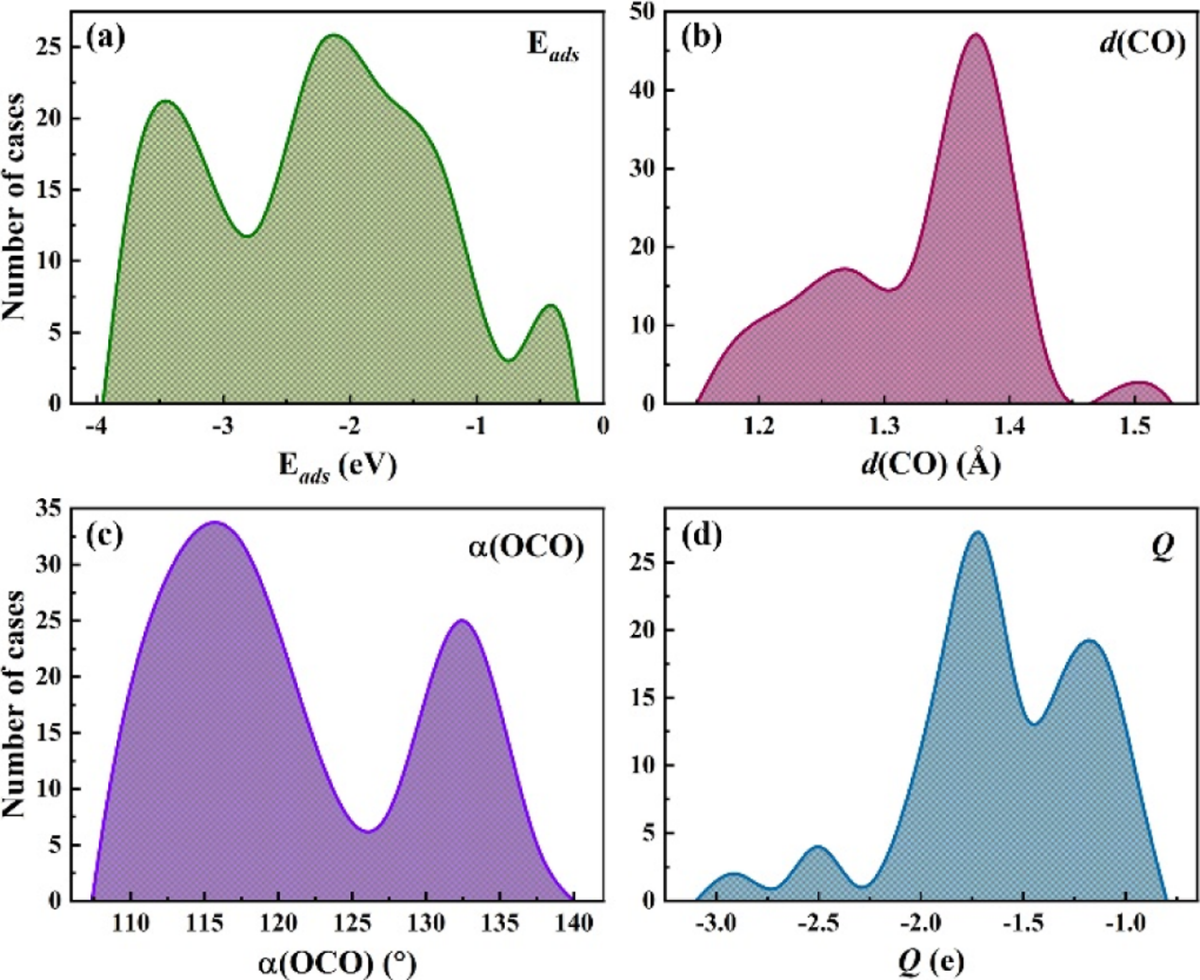
Density distribution of (*a*) CO_2_*E*_ads_, (b) C–O bond distance, *d*(CO), (c) CO_2_ angle, α(OCO), and (d) Bader charges, *Q*, for the complete set of data of 114 MXene cases.

At first, we evaluated these features in a descriptive fashion, showing fringe limits in the data set and distribution; see Figure 3. A quick inspection reveals that the distribution of features is not uniform for none of the target properties. For instance, *E*_ads_ shows three peaks, one close to *ca.* −3.5 eV, another around −2.1 eV, and a small peak close to −0.4 eV. According to the *Sabatier* principle, moderate adsorption energies —neither too weak nor too strong— would provide the better catalytic performances, but, in our case, an activated CO_2_ molecule getting bent and negatively charged often implies strong adsorption energies, suggesting that a surplus of energy is required for a reaction to occur when using adsorbed CO_2_. In any case, among all the studied MXenes, only 6.84% —6 out of 114— of the *E*_ads_ are below −1.0 eV, which indicates overall a strong CO_2_ chemisorption over the studied MXenes.

The previous property is accompanied by reduced angles and elongated C–O bonds, indicators of the CO_2_ activation.^58^ In the latter case, they are concentrated at 1.37 Å, which is 0.20 Å larger than the CO_2_ distance in vacuum of 1.17 Å, with a smaller peak at 1.27 Å, and few cases with bond lengths larger than 1.5 Å, like Cr_2_C with a *d*(CO) of 1.54 Å. When it comes to molecular angles, there are two main peaks around 116 and 132°. Notice thus that all the studied cases imply a bent CO_2_, with angles ranging from 112 to 140°. The increase in both C–O bond elongation and CO_2_ bending is consistent with a charge transfer from the surface to the adsorbed molecule.^59^ Thus, the Bader charge of the adsorbed CO_2_ is also a potential indicator of activation, where the average *Q* is found to be −1.59 *e*, with a minimum and maximum value of −2.98 and −0.83 *e*, respectively, and a significant peak around −1.1 *e*.

**Figure 4:**
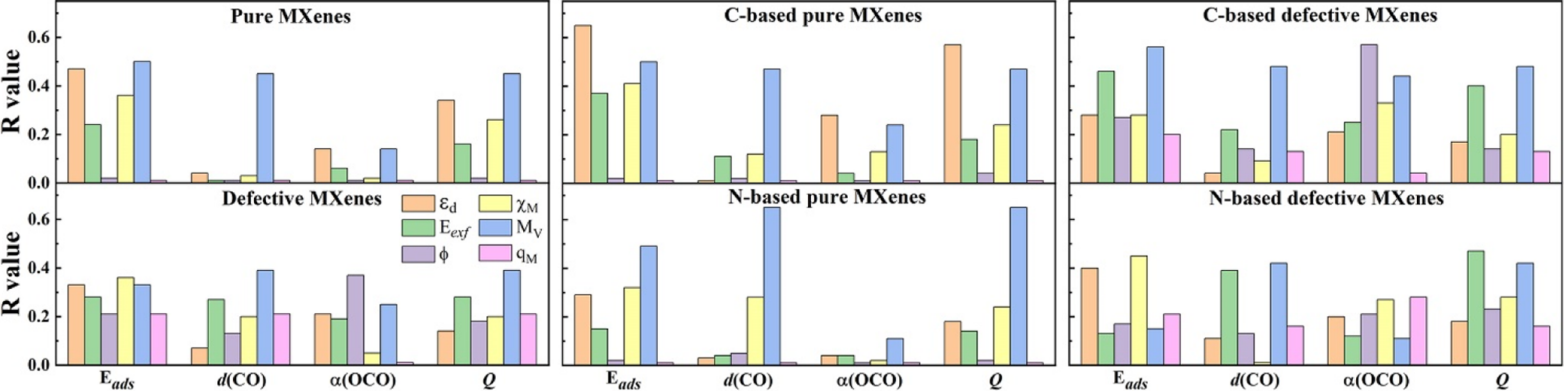
Regression coefficients, *R*, for the linear correlation between the target properties, *E*_ads_, *d*(CO), α(OCO), and *Q*, and primary features, ε_d_, *E*_exf_, ϕ, χ_M_, *M*_V_, and *q*_M_.

To understand the efficiency of a catalyst, one requires descriptors that correlate with the catalyst performance. Hence, for a practical use, the selected primary features or descriptors should be much facile to evaluate when compared with that of the target properties and, whenever possible, connect with chemical intuition-derived concepts. Thus, for a fruitful comparison of unique fingerprints, we have considered 18 primary features aimed to characterize the local environment of the adsorption sites, chosen among the properties of pristine MXenes, but also including features from the atoms comprising the MXene. These primary features are rapid to obtain, unique, and easily accessible. Typically, since the binding energies scale linearly with the *d*-band filling, the adsorption strength could be linked to the TM *d*-band energy distribution. Figure S4 displays the linear correlations between the target properties; *E*_ads_, *d*(CO), α(OCO), and *Q*, and primary features of MXenes, including some of the best performing or alleged descriptors in the literature, such as the *d*-band center, ε_d_, the exfoliation energy, *E*_exf_, the work function, ϕ, the metal electronegativity, χ_M_, the valence electron number of a metal atom, *M*_V_, and Bader charge of surface metal atom, *q*_M_, along with the regression coefficients *R*. For a better understanding, the *R* values of the aforementioned descriptors are provided separately for pure and defective MXenes and summarized in Figure 4, also regarding C- and N-based MXenes separately.

As seen in Figure 4, for both pure and defective MXenes, *E*_ads_ shows better linear trends with the primary features, while *d*(CO) and α(OCO) show poor correlations when compared with other target properties. In the case of pure MXenes with varying thicknesses, the detailed analysis demonstrates that the *R* value of *E*_ads_ as a function of ε_d_ improves by increasing it. For defective systems, the *R* value is smaller for single vacancies; *V*_M_ and/or *V*_X_, while the *R* score increases in the case of double vacancies, *i.e.*, *V*_MX_. Interestingly, the *R* value of several primary features exceeds that of the *d*-band center. For instance, in all cases, *M*_V_ shows better scaling relations among the other descriptors. It should be noted that the *d*-band center is quite a universal descriptor for *E*_ads_ of different adsorbates at transition meal surfaces representing catalyst models. However, there are several signatures that the *d*-band center itself is not an adequate descriptor for more complex compounds.^60−63^ On the other hand, *q*_M_ shows very small regression coefficients, indicating that the target properties exhibit poor correlations with the primary features. Notably, *M*_V_ and ε_d_ appear as the top two descriptors, independently establishing the relationship with the target properties. However, we were unable to establish a better relationship with target properties using simply several single descriptors, which requires integration of multiple descriptors to reach a more accurate description. Therefore, these insufficient correlations prompted us to build a predictive ML model through combination of primary features that could resemble the contribution of each feature individually to the model.

Thus, using primary features as input variables, we evaluated various ML models, including MLR, DTR, and RFR methods using our database. Their MAE together with the standard deviation, σ, are shown in Table 1. In the case of *E*_ads_, the MAE values are found to be 0.49 ± 0.06, 0.53 ± 0.10, and 0.45 ± 0.06 eV for MLR, DTR, and RFR, respectively. Notice that such errors are around double the typical DFT accuracy of *ca.* 0.2 eV and are still too large, especially when predicting cases with an *E*_ads_ weaker than −1 eV. However, for the majority of MXene cases, the accuracy is already enough for a rapid screening, being the most of the cases between −1 and −4 eV; see Figure 1. For all the combinations of descriptors, the RFR model showed better performance than MLR and DFR models. Typically, feature importance estimates the weightage of a particular descriptor, thereby revealing the most relevant features for predicting the target properties by understanding the direct chemical insights. Especially for catalytic materials,^30,64−67^ analyzing primary features is meaningful and interesting to predict the correlation between the target properties and the descriptors form view point of underlying physics and chemistry.

**Table 1:** MAE ± Standard Deviation, σ, of *E*_ads_, *d*(CO), α(OCO), and *Q* Using MLR, DTR, and RFR ML Regressors, as Well as RFR RUF, and HT over RUF

ML model	*E*_ads_/eV	*d*(CO)/Å	α(OCO)/deg	*Q*/*e*
MLR	0.49 ± 0.06	0.05 ± 0.01	5.4 ± 0.7	0.22 ± 0.04
DTR	0.53 ± 0.10	0.06 ± 0.01	5.5 ± 1.3	0.27 ± 0.05
RFR	0.45 ± 0.06	0.04 ± 0.01	4.9 ± 0.6	0.21 ± 0.03
RUF	0.43 ± 0.13	0.04 ± 0.01	4.9 ± 0.6	0.21 ± 0.03
HT	0.42 ± 0.06	0.04 ± 0.01	4.8 ± 0.8	0.20 ± 0.03

For CO_2_*E*_ads_, the top five important features are the group number of the metal atom, *G*_M_, χ_M_, *M*_V_, ε_d_, and *E*_exf_. In the case of *d*(CO), α(OCO), and *Q*, the combinations of (ε_d_, *E*_exf_, ϕ, *q*_M_, and χ_M_), (χ_M_, ε_d_, *q*_M_, ϕ, and number of d electrons, *Nd*_M_), and (*M*_V_, χ_M_, ϕ, ε_d_ and *Nd*_M_) were rendered as the top features. Among them, *G*_M_, χ_M_, *M*_V_, and *Nd*_M_ are tabulated chemical element properties, while ε_d_, *E*_exf_, *q*_M_, and ϕ are DFT computed descriptors. To further understand the importance of precise features that correlate the target properties, it is necessary to remove the descriptors that are less relevant in minimizing the MAE. It should also be noted that an excessive number of features may lead to high prediction bias and low training efficiency.^68^ To alleviate this issue, the feature dimension is reduced by employing the leave-one-out approach. Using this method, we eliminate unwanted features by evaluating their impact on the test set MAE. After shortlisting the descriptors according to the leave-one-out approach, HT was performed over RFR by employing cross-validation on various combinations of parameters. Although removing unnecessary features (RUF) and HT exhibited comparable performance, the latter marginally outperformed the former in terms of least MAE.

**Figure 5:**
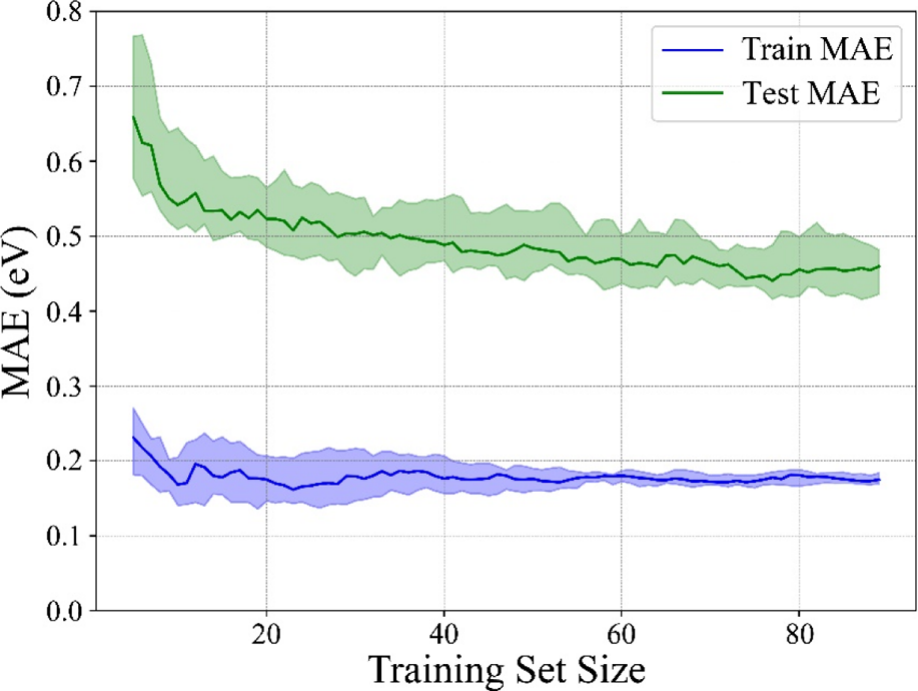
MAE evolution for the training (blue) and test set (green) *versus* training size for the prediction of *E*_ads_ using the HT of RFR ML algorithm. Shaded regions define the standard deviation limits.

As per the size of the data set, Figure 5 shows the *E*_ads_ MAE decay with respect to training set size; in other words, the learning curve, regarding that training set considers randomly selected 80% of the samples, while the test set comprises the remaining 20%. For a better analysis, a cross-validation procedure with 100 shuffle splits was carried out, as done in previous analysis, where average MAE is shown in Figure 5, with areas denoting the standard deviations.^69^ Notice on the training set that RFR MAE decay is rather good, 0.16 ± 0.01 eV, rapidly below the 0.2 eV DFT accuracy limit, and especially with an almost negligible standard deviation when having more than *ca.* 60 samples. Still, the decay of the test set is more pronounced, with larger standard deviations; see Table 1, and with the open question whether the evolution would remain stuck or would still descend when increasing the number of points of the data set. Alternatively, the reached plateau may be indicative of the existence of other descriptors, here not accounted for, which could be critical in improving the accuracy. Similar MAE evaluation is found for *d*(CO), α(OCO), and *Q* in Figures S5–S7 of the Supporting Information.

**Figure 6:**
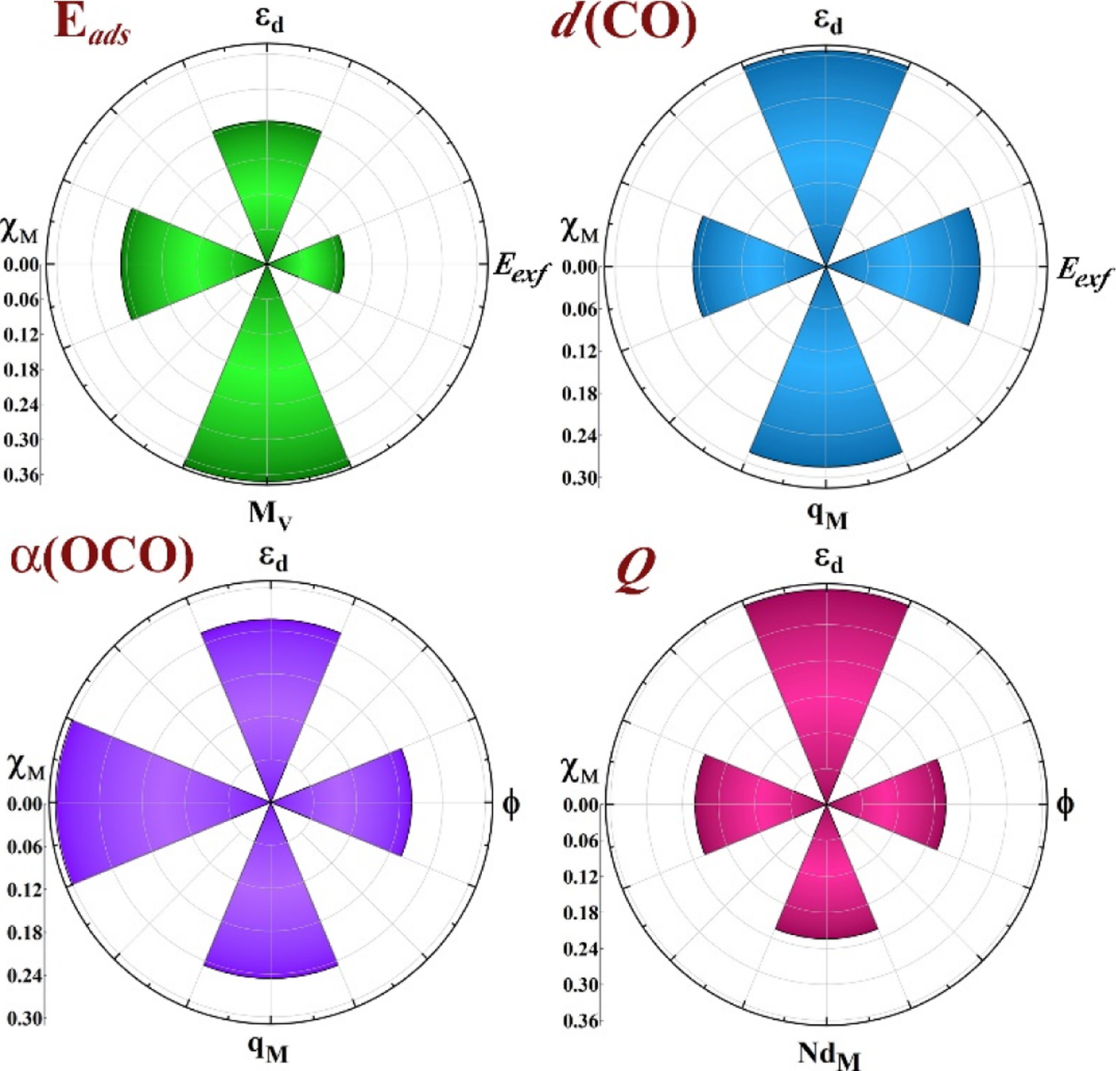
Feature importance of top four descriptors for *E*_ads_, *Q*, *d*(CO), and α(OCO).

The HT of RFR further improved the accuracy of the model for *E*_ads_ by reducing the test set MAE to 0.42 ± 0.06 eV. Indeed, estimations on the MAE on the HT of RFR ML using a limit training set of 113 points, and evaluated on the remaining test point, provides slightly better accuracies of 0.15 and 0.40 eV for training and test sets, respectively, over 114 developed ML models, signaling the convergence of the accuracy over the data set. The top four descriptors listed by the RFR model for *E*_ads_ are the combination of two features of the TM chemical elements, χ_M_ and *M*_V_, plus two other computed for the MXenes, ε_d_ and *E*_exf_; see Figure 6, highlighting how important surface metal atoms are and how important is their placement within the MXene arrangement. It should also be noted that the choice of features introduces biasing, but at the same time, favors to counterbalance the overfitting, since we narrow their choice to sensible parameters that have been correlated to the sought, target properties, according to the literature. In the case of *d*(CO) and α(OCO), the MAE of the testing set is rather good as well, which are found to be 0.04 ± 0.01 Å and 4.84 ± 0.78°, respectively, essentially four times larger than chemical accuracy limits of 0.01 Å and 1°, respectively. For *Q*, there is a slight decrease in the prediction performance of RFR using HT; from 0.21 ± 0.03 to 0.20 ± 0.03 *e*, when compared to RUF. To reinforce the employed methodology, we have also compared our results using the recursive feature elimination (RFE)^70^ method to filter the descriptors with extreme asymmetry (skewness) and with low/zero variance for recognizing more suitable smaller subset of features. As shown in Table S1 of the Supporting Information, the leave-one-out approach outperforms the RFE method by providing better predictive mean absolute errors. Finally, notice in Figure 6 that ε_d_ and χ_M_ are common descriptors of all the explored properties, while others such as ϕ, *q*_M_, and *E*_exf_ are also common to a couple of properties, while *M*_V_ and *Nd*_M_ are only important to *E*_ads_ and *Q*, respectively.

Notice that the abovementioned ML models work irrespective of MXenes with or without vacancies, and for either C- or N-based MXenes, at variance with linear relationships; see Figure S4 of the Supporting Information, highlighting the versatility of the ML approach. Inspecting the descriptor weights in Figure 6, the ranking already states how ε_d_ and χ_M_ are determinant in CO_2_ activation, where the larger the ε_d_, the stronger the bonding is, as expected from the d-band model.^19^ Aside, the smaller the metal electronegativity, χ_M_, the stronger the *E*_ads_, fully physically understandable given the coulombic contribution of the bond of negatively charged CO_2_ with positively charged surface metal atoms in the MXenes; see Figure S4 in the Supporting Information. In any case, the weights of these two primary features are different for the different properties, *e.g.*, ε_d_ weights are 30 and 35% for *Q* and *d*(CO), respectively, while for *E*_ads_, actually χ_M_ and ε_d_ have similar importance values of 25%. Other secondary features can be rationalized as well; for instance, the CO_2_ charge *Q* also pretty much affects the molecular angle, α(OCO), and is influenced by a smaller workfunction, ϕ, which succinctly implies an easier MXene→CO_2_ charge transfer. *E*_exf_ affects the bond strengths, and so, the larger the *E*_exf_, the smaller the CO_2_ adsorption energy and the less elongated becomes *d*(CO). As far as geometries are concerned, *d*(CO) and α(OCO) seem to be slightly influenced as well by *q*_M_, so that the larger the charge, the smaller the α(OCO) angle and the longer the *d*(CO), stabilizing the negatively charged CO_2_. Finally, the number of valence electrons and the number of d electrons, somehow related, affect the *E*_ads_ and the amount of transferred *Q*, in the sense that the smaller the number of valence electrons, and so, of d electrons, the stronger the *E*_ads_ and the more charge transferred, also in line with higher ε_d_. By identifying these descriptors, we have gained a deeper insight of the fundamental properties governing CO_2_ activation on the studied MXene surfaces, which can ultimately be used to design and optimize MXene-based compounds for CO_2_ storage or conversion applications. Thus, the ML tools allowed us to name which factors govern the CO_2_ activation, and which importance they have, which are properties to have in mind when inspecting other MXenes for CO_2_ storage or usage selected processes. For example, from the descriptor weights in Figure 6, when one would seek for CO_2_*E*_ads_ of −1 eV or weaker, one should pay attention to the *M*_V_, ε_d_, χ_M_, and *E*_exf_ descriptors; which is in line with the trends evaluated in Figure S4 of the Supporting Information; one would seek the MXene materials with ε_d_ below −1 eV, while having an *E*_exf_ above 3.25 J·m^–2^, a metal electronegativity of the metal, χ_M_, above 1.5, and a minimum number of 6 *e* valence electrons of the metal, *M*_V_. Moreover, the coefficient of determination analysis; see heat map in Figure S8 of the Supporting Information, demonstrates that the reduced set of features is sufficient for capturing the complex interactions influencing the *E*_ads_, *d*(CO), α(OCO), and *Q*, with no significant linear correlation among the found descriptors.

## 4 Conclusions

In summary, we have developed a ML prediction scheme to unearth the potential indicators for CO_2_ activation on MXenes with the accessible properties of the pristine materials and of the atoms they are composed of. Three different ML algorithms were trained, where the hyperparameters tuning of RFR improved the accuracy of the model for *E*_ads_, reducing the test set MAE to 0.42 ± 0.06 eV when compared with that of the conventional RFR model, while the training set MAE was 0.16 ± 0.01 eV. The high ranking of the *d*-band center, ε_d_, and surface metal electronegativity, χ_M_, is highlighted for *E*_ads_, but also for other activation properties, including CO_2_ charge, *Q*, *d*(CO) bond length elongation, and molecular angle α(OCO) bending. These primary features are followed by valence and d electron numbers, *M*_V_ and *Nd*_M_, and also MXene workfunctions, ϕ, exfoliation energies, *E*_exf_, and surface metal charges, *q*_M_, features, predicting the activation of CO_2_, demonstrating the importance of such surface properties, and serving as a guide to select or search certain MXene materials for CO_2_ activation and/or use applications. Overall, the discovery of key descriptors for CO_2_ activation highlights the importance of ML strategies for accelerating the catalyst materials design and development by significantly extracting the information from a limited set of MXene database.

## Supporting Information Available

Description of the employed ML algorithms, including the MLR, the decision tree regressor, and the random forest regressor, graphical insights on how *E*_exf_ is calculated, lists of target properties and primary features, all linear correlations between target properties and primary features, distinguishing bare and defective MXenes, ML model learning curves, MAE when using the leave-one-out or the RFE procedures, coefficient of determination heat maps for the employed features, and references (PDF)
