# Supplementary material for: Machine Learning-Driven Discovery of Key Descriptors for CO2 Activation over Two-Dimensional Transition Metal Carbides and Nitrides
Source: ACS Appl Mater Interfaces. 2023 Jun 19;15(25):30117–26. doi: 10.1021/acsami.3c02821 (PMC10316327; doi:10.1021/acsami.3c02821)
Supplement: Supplementary file 1 — am3c02821_si_001.pdf [file am3c02821_si_001.pdf]

Machine Learning-Driven Discovery of Key Descriptors for CO_2 Activation over Two-Dimensional Transition Metal Carbides and Nitrides

B. Moses Abraham,[†,‡] Oriol Piqué,[‡] Mohd Aamir Khan,[†§] Francesc Viñes,[*,‡] Francesc Illas[‡] and Jayant K. Singh[*†§]

[†] *Department of Chemical Engineering, Indian Institute of Technology Kanpur, Kanpur-208016, India*

[‡] *Departament de Ciència de Materials i Química Física, Institut de Química Teòrica i Computacional (IQTCUB), Universitat de Barcelona, c/ Martí i Franquès 1-11, 08028 Barcelona, Spain*

[§] *Prescience Insilico Private Limited, Bangalore 560049, India*

∗e-mails: francesc.vines@ub.edu, jayantks@iitk.ac.in

S1. Machine learning regression algorithms

Machine learning (ML) models optimize the performance of a given assignment by utilizing past experience. Typically, ML can be divided into three categories: *i*) supervised, which uses the same principles as a standard fitting procedure, and data labeling is imposed by the user, *ii*) unsupervised, finding patterns in unlabeled data, and *iii*) semi-supervised, where algorithm is given with both labeled as well as unlabeled data. In the Materials Science discipline, supervised learning is a highly utilized category of ML. Here, the form (x,y) is used, where x (matrix of features) belongs to a system (*e.g.*: composition and materials structure) to be designed and the feature of the system is described by each element in that row; while y corresponds to a vector of target properties to be designed. The x generally starts in the form of simple description and the corresponding features are generated in the numerical form. The x and y are related as $y = \mathrm{F}(x) + \varepsilon$, where ε represents noise term and we construct a ML model for $\mathrm{F}(x)$. From a vector of descriptor (x^*), one can predict corresponding single target value *via* ML model, $\hat{y}^* = \hat{F}(x^*)$. Typically, $\hat{F}$ is described by its model type, *e.g.* linear regression or decision tree regression, parameters (coefficients of linear terms), and hyper-parameters (matrix inversion). The *sklearn* Python library[1] is employed to settle and optimize all the ML algorithms used in the present work.

S2. Multivariate linear regression

Multivariate linear regression (MLR) is a supervised modeling algorithm capable of interrogating the interactions between catalyst and substrate by understanding the specific physicochemical features. These features are typically captured by a set of descriptors, generally divided between electronic and steric descriptors with a numerical value computed from the optimized structure of a catalyst. In a simple scenario, MLR extends the fundamental idea of linear regression model that involves only one response variable to many variables.

S3. Decision tree regressor

Decision tree regressor (DTR) is a supervised ML model that can handle both classification and regression problems. DTR is a better option when the linear models display low accuracy, which is usually easy to understand and requires lesser data-cleaning because the results are not affected by the multi-collinearity. The model is constructed in the form of a tree structure using recursive partitioning of the data set. In the tree structure, the root of the tree is the topmost node corresponding to the best predictor, and each branch indicates a possible decision. The

DTR model breaks down the input data into smaller subsets of different regions by establishing consecutive splitting rules by solving "if-else" problems, as executed in each internal node the tree. The first split contains maximum information and this process continues until information obtain is approximately close to zero or no more information can be gained. The predicted result at leaf nodes is the output of the algorithm for all observations in that node. Unnecessary complex trees can be avoided by setting the maximum tree depth and minimum samples needed at a leaf node. This model defines best questions by understanding possible relationships between the data and the target variable to produce meaningful output.

To better understand the complexity of the above-mentioned model, the implementation of DTR algorithm is demonstrated with an example, where the recursive data-splitting process is explained using Figures S1 and S2. Under the simplifying assumption, the target variable y (*e.g.* E_{ads}) depends on two background characteristics, x_1 and x_2 (such as ε_d and ϕ). From the scattering plot x_1 *vs.* x_2, see Fig. S1, the first algorithmic split occurred at x_2 greater than 40, indicating that the two data samples generated through this split are relatively more pure when compared to initial data. The next split questions whether x_1 value is greater than 6 for x_2 values larger than 40, or x_1 value being greater than 9 when x_2 is smaller than 40, see Figure S2. The algorithm stops proceeding further when the splitting data does not obtain any purified data samples. This process leads to four leaves with different $\bar{y}$ average values of the y_i values contained in each sectioned region. Thus, under a built tree in DTR, an averaged $\bar{y}$ value is predicted based on descriptors x_1 and x_2.

Figure S1. Schematic representation of DTR data splitting. Here dashed lines indicate the variable splitting decisions learned by the model. A, B, C and D represent the split regions, from which averaged $\bar{y}$ values are taken from, while a_i are values for the node questions.

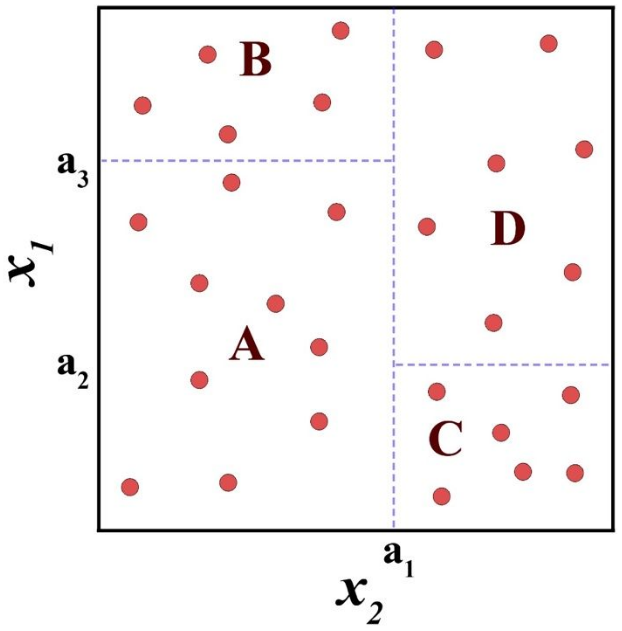

Figure S2. Schematic representation of the DTR shown in Fig. 1.

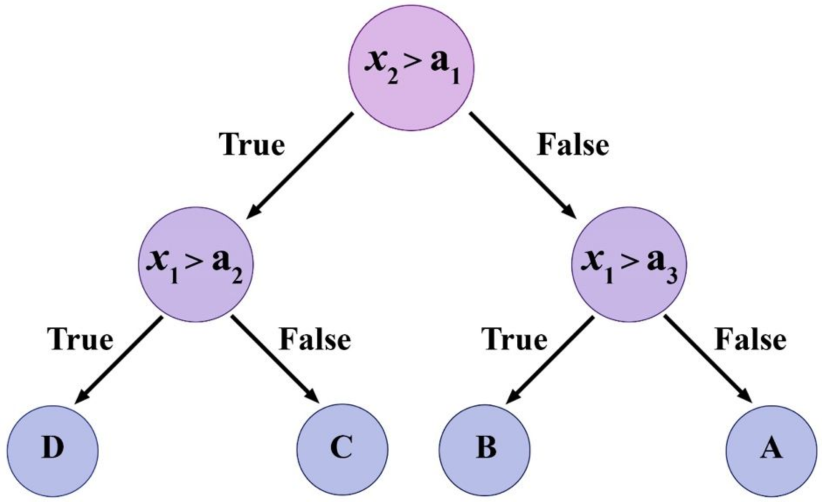

S4. Random forest regressor

The above commented DTR model can leads to over-fitting problems, which can be solved by employing the random forest regression (RFR) algorithm. It is an ensemble approach where multiple decisions are fused to obtain better predictive performance. RFR uses random feature selection in tree induction and bootstrap samples of the training data. The principle of the model is based on building a training set and constructing a predictor tree that connects the observation of a variable to get stable and accurate prediction of the target. Each tree in the ensemble generate an output based on the molecular/structural properties or descriptors, and results from all trees are aggregated to give a final average prediction. From the above-mentioned background characteristics x_1 and x_2, different average values are produced for $I = 1 - N$, where N indicates number of trees of the forest (*n_estimators*). Thus, the output is simply the average over the expected values on various N trees.

The RFR becomes DTR for $i = 1$, which indicates better accuracy for more number of trees. This procedure offer some unique features by reducing over-fitting, including measures of variable importance and built-in performance assessment,[2,3] which makes it appropriate for tasks that exclusively depends on quantitative structure-activity relationship (QSAR).[4]

S5. MAX unit cell and the isolated MXene

Figure S3. Side views of (a) MAX crystal cell and (b) its derived MXene slab cell. Pink, blue, and grey color balls denote M, A, and X elements, respectively.

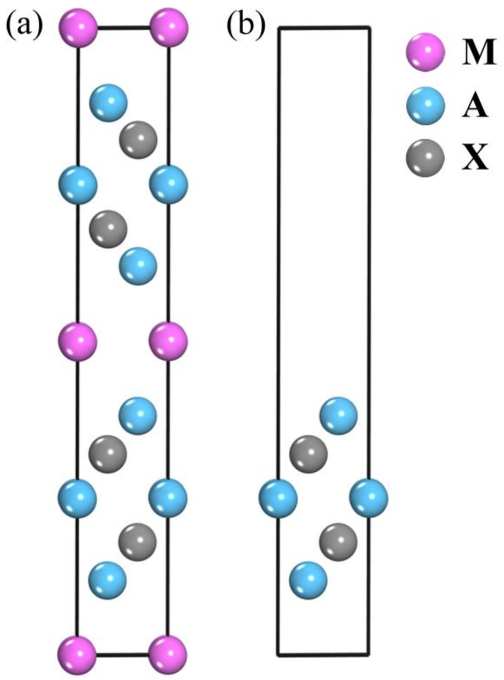

S6. List of target properties

	**Target Properties**
$\boldsymbol{E_{ads}}$	CO_2 adsorption energy
**α(OCO)**	CO_2 molecular angle
***d*(CO)**	CO_2 average bond length
$\boldsymbol{Q}$	CO_2 Bader charge

S7. List of primary features or descriptors

	Primary Features
ε_d	*d*-band center
E_{exf}	Exfoliation energy
ϕ	Work function
**X**	Carbon or Nitrogen
**L**	Layer thickness
P_X	Periodic number of the X atom
P_M	Periodic number of the metal M atom
G_X	Group number of the X atom
G_M	Group number of the M atom
N_X	X atomic number
N_M	M atomic number
Np_X	Number of X *p* electrons
Nd_M	Number of M *d* electrons
V_X	Number of X valence electrons
V_M	Number of M valence electrons
χ_X	Electronegativity of X atom
χ_M	Electronegativity of M atom
Q_M	Bader charge of surface M atoms

S8. Individual linear regressions

Figure S4. Linear correlations between the target properties; E_{ads}, d(CO), α(OCO), and Q, given in eV, Å, º, and e, respectively; and primary features, ε_d, E_{exf}, ϕ, χ_M, M_V, and q_M, given in eV for both ε_d and ϕ, $J{\cdot}m^{-2}$ for E_{exf}, e for M_V and q_M, while χ_M is dimensionless. Each adjustment displays the regression coefficients, R, distinguishing cases pure and defective MXenes, C- or N-based ones, having V_M, V_X or V_{MX} vacancies, or different $M_{n+1}X_n$ widths.

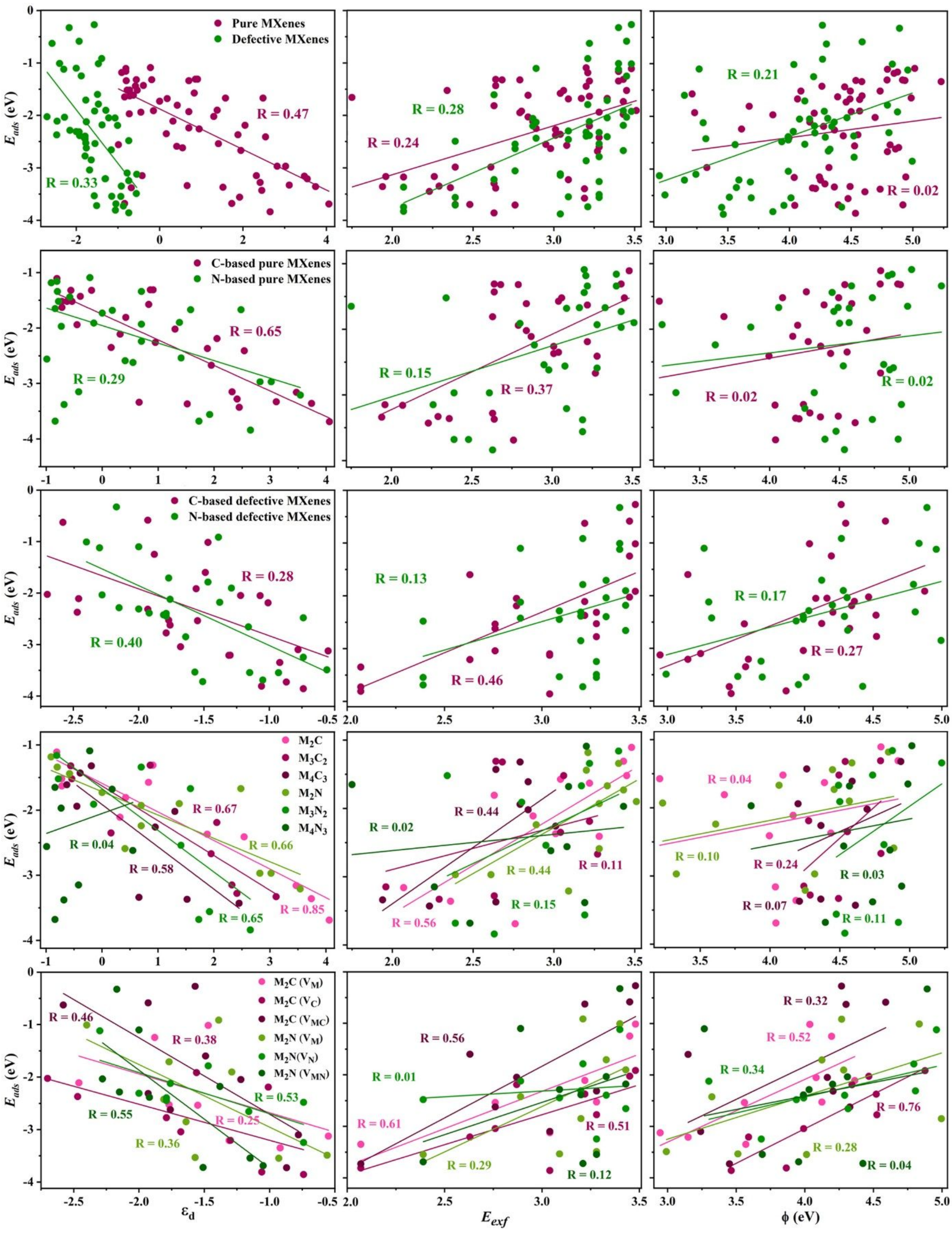
Pure MXenes
Defective MXenes
C-based pure MXenes
N-based pure MXenes
C-based defective MXenes
N-based defective MXenes
E_{ads} (eV)
ε_d
E_{exf}
ϕ (eV)

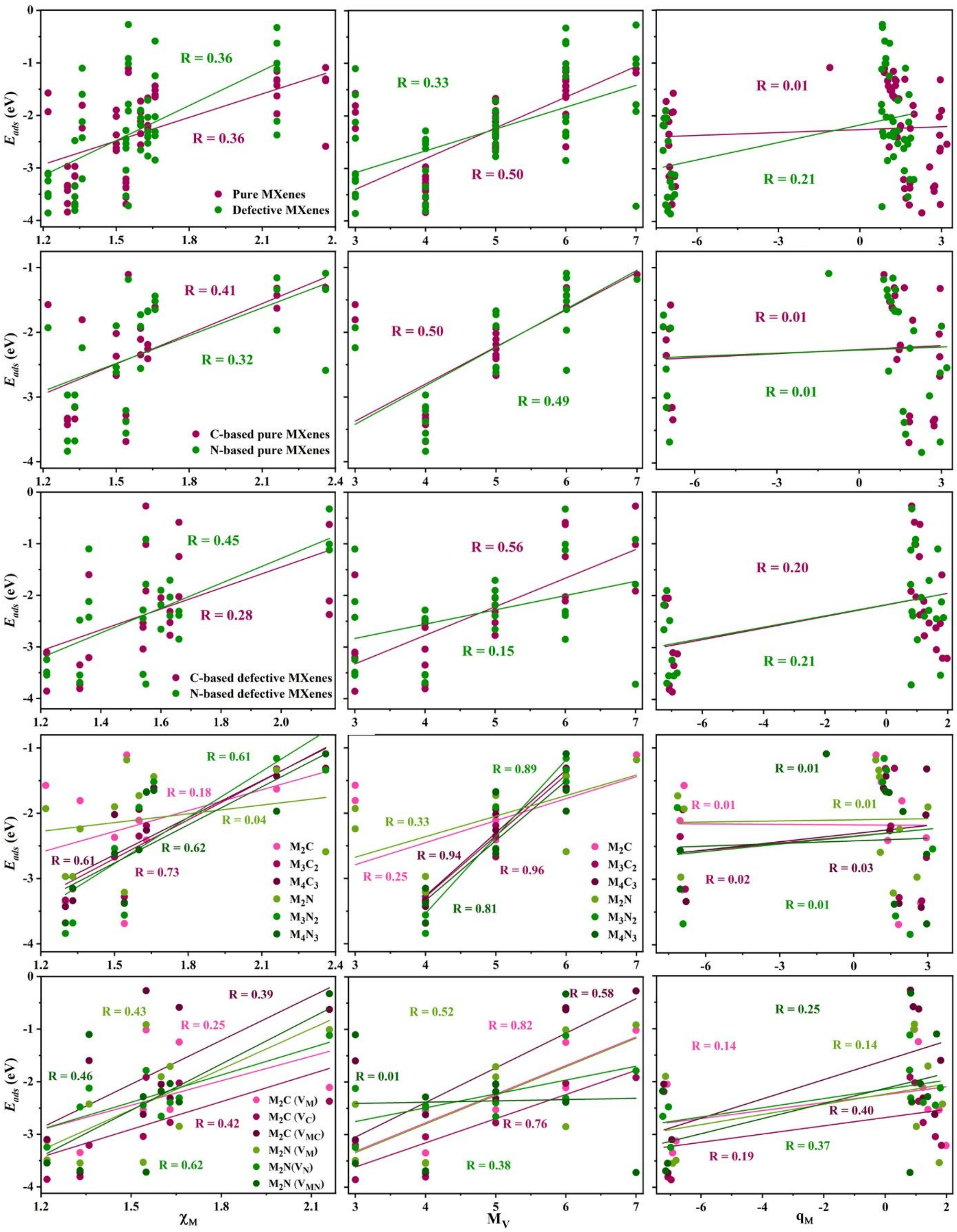

R = 0.36
R = 0.36
Pure MXenes
Defective MXenes
R = 0.33
R = 0.50
R = 0.01
R = 0.21
R = 0.41
R = 0.32
C-based pure MXenes
N-based pure MXenes
R = 0.50
R = 0.49
R = 0.01
R = 0.01
R = 0.45
R = 0.28
C-based defective MXenes
N-based defective MXenes
R = 0.56
R = 0.15
R = 0.20
R = 0.21
R = 0.61
R = 0.18
R = 0.04
R = 0.62
R = 0.61
R = 0.73
M2C
M3C2
M4C3
M2N
M3N2
M4N3
R = 0.89
R = 0.33
R = 0.94
R = 0.25
R = 0.96
R = 0.81
R = 0.01
R = 0.01
R = 0.01
R = 0.03
R = 0.02
R = 0.01
R = 0.39
R = 0.43
R = 0.25
R = 0.46
R = 0.42
R = 0.62
M2C (VM)
M2C (VC)
M2C (VMC)
M2N (VM)
M2N(VN)
M2N (VMN)
R = 0.52
R = 0.58
R = 0.82
R = 0.01
R = 0.76
R = 0.38
R = 0.25
R = 0.14
R = 0.14
R = 0.40
R = 0.37
R = 0.19
Eads (eV)
χM
MV
qM

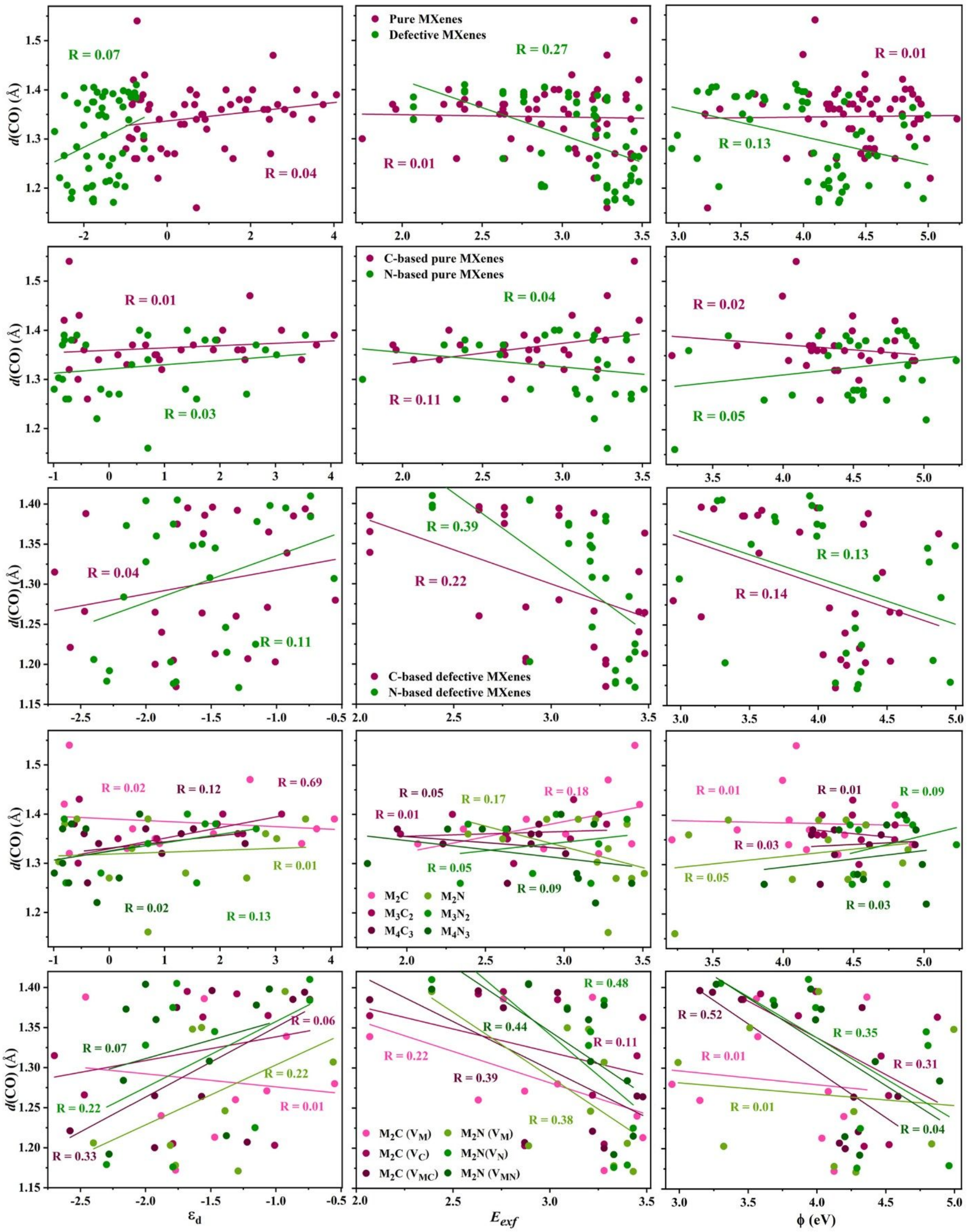
Pure MXenes
Defective MXenes
R = 0.07
R = 0.04
R = 0.27
R = 0.01
R = 0.01
R = 0.13
C-based pure MXenes
N-based pure MXenes
R = 0.01
R = 0.03
R = 0.04
R = 0.11
R = 0.02
R = 0.05
R = 0.04
R = 0.11
R = 0.39
R = 0.22
C-based defective MXenes
N-based defective MXenes
R = 0.13
R = 0.14
R = 0.02
R = 0.12
R = 0.69
R = 0.01
R = 0.02
R = 0.13
R = 0.05
R = 0.01
R = 0.17
R = 0.18
R = 0.05
R = 0.09
M_2C
M_3C_2
M_4C_3
M_2N
M_3N_2
M_4N_3
R = 0.01
R = 0.01
R = 0.09
R = 0.03
R = 0.05
R = 0.03
R = 0.07
R = 0.06
R = 0.22
R = 0.22
R = 0.01
R = 0.33
R = 0.48
R = 0.44
R = 0.11
R = 0.22
R = 0.39
R = 0.38
M_2C (V_M)
M_2C (V_C)
M_2C (V_{MC})
M_2N (V_M)
M_2N(V_N)
M_2N (V_{MN})
R = 0.52
R = 0.35
R = 0.01
R = 0.31
R = 0.01
R = 0.04
d(CO) (Å)
ε_d
E_{exf}
ϕ (eV)

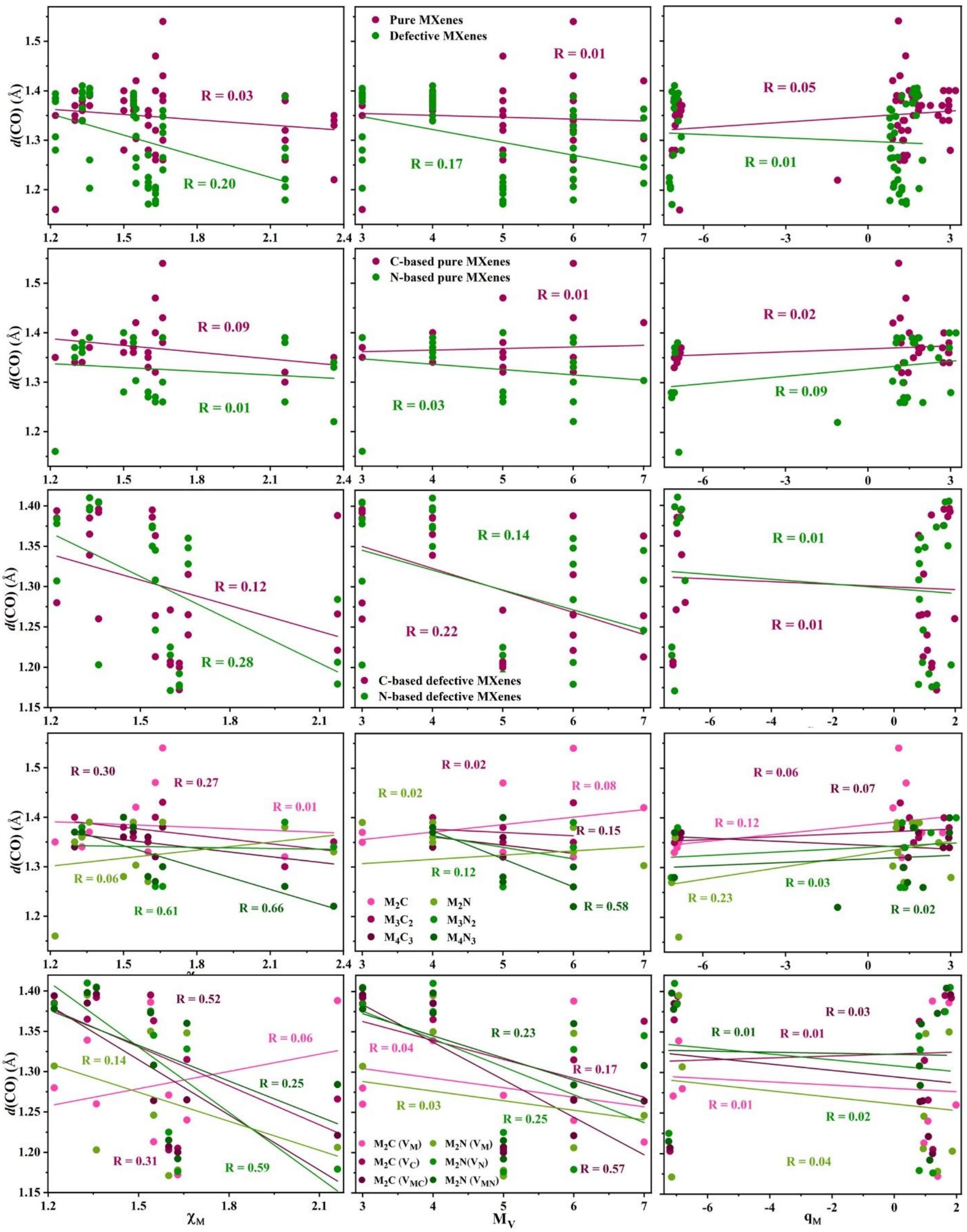
Pure MXenes
Defective MXenes
C-based pure MXenes
N-based pure MXenes
C-based defective MXenes
N-based defective MXenes
M2C
M3C2
M4C3
M2N
M3N2
M4N3
M2C (VM)
M2C (VC)
M2C (VMC)
M2N (VM)
M2N(VN)
M2N (VMN)
d(CO) (Å)
χM
MV
qM
R = 0.03
R = 0.20
R = 0.01
R = 0.17
R = 0.05
R = 0.01
R = 0.09
R = 0.01
R = 0.01
R = 0.03
R = 0.02
R = 0.09
R = 0.12
R = 0.28
R = 0.14
R = 0.22
R = 0.01
R = 0.01
R = 0.30
R = 0.27
R = 0.01
R = 0.06
R = 0.61
R = 0.66
R = 0.02
R = 0.02
R = 0.08
R = 0.15
R = 0.12
R = 0.58
R = 0.06
R = 0.07
R = 0.12
R = 0.23
R = 0.03
R = 0.02
R = 0.52
R = 0.06
R = 0.14
R = 0.25
R = 0.31
R = 0.59
R = 0.04
R = 0.23
R = 0.03
R = 0.17
R = 0.25
R = 0.57
R = 0.03
R = 0.01
R = 0.01
R = 0.01
R = 0.02
R = 0.04

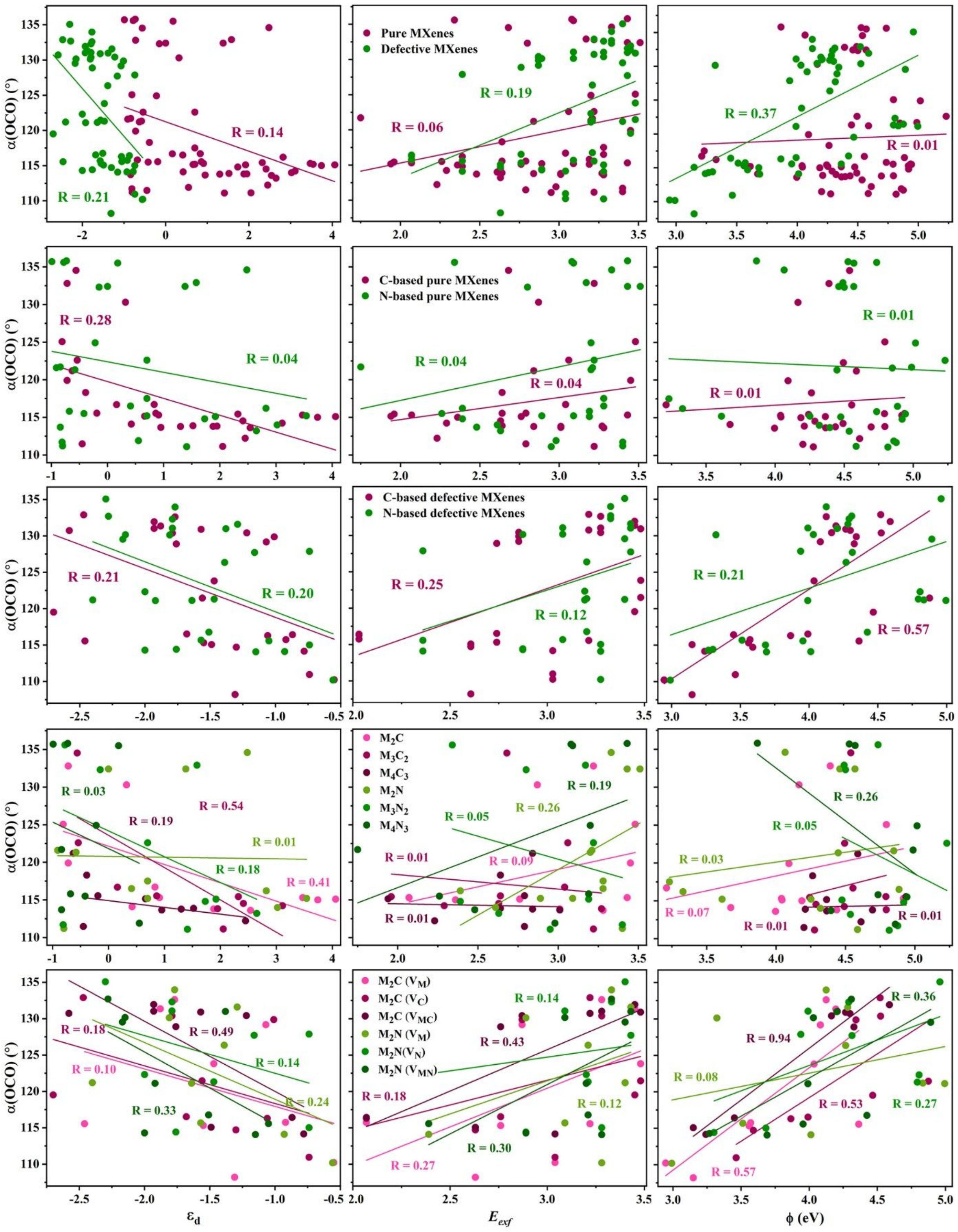
α(OCO) (°)
Pure MXenes
Defective MXenes
R = 0.21
R = 0.14
R = 0.06
R = 0.19
R = 0.37
R = 0.01
C-based pure MXenes
N-based pure MXenes
R = 0.28
R = 0.04
R = 0.04
R = 0.04
R = 0.01
R = 0.01
C-based defective MXenes
N-based defective MXenes
R = 0.21
R = 0.20
R = 0.25
R = 0.12
R = 0.21
R = 0.57
M2C
M3C2
M4C3
M2N
M3N2
M4N3
R = 0.03
R = 0.19
R = 0.54
R = 0.01
R = 0.18
R = 0.41
R = 0.05
R = 0.26
R = 0.19
R = 0.01
R = 0.09
R = 0.01
R = 0.26
R = 0.05
R = 0.03
R = 0.07
R = 0.01
R = 0.01
M2C (VM)
M2C (VC)
M2C (VMC)
M2N (VM)
M2N(VN)
M2N (VMN)
R = 0.18
R = 0.49
R = 0.10
R = 0.14
R = 0.33
R = 0.24
R = 0.14
R = 0.43
R = 0.18
R = 0.12
R = 0.30
R = 0.27
R = 0.94
R = 0.36
R = 0.08
R = 0.53
R = 0.27
R = 0.57
εd
Eexf
ϕ (eV)

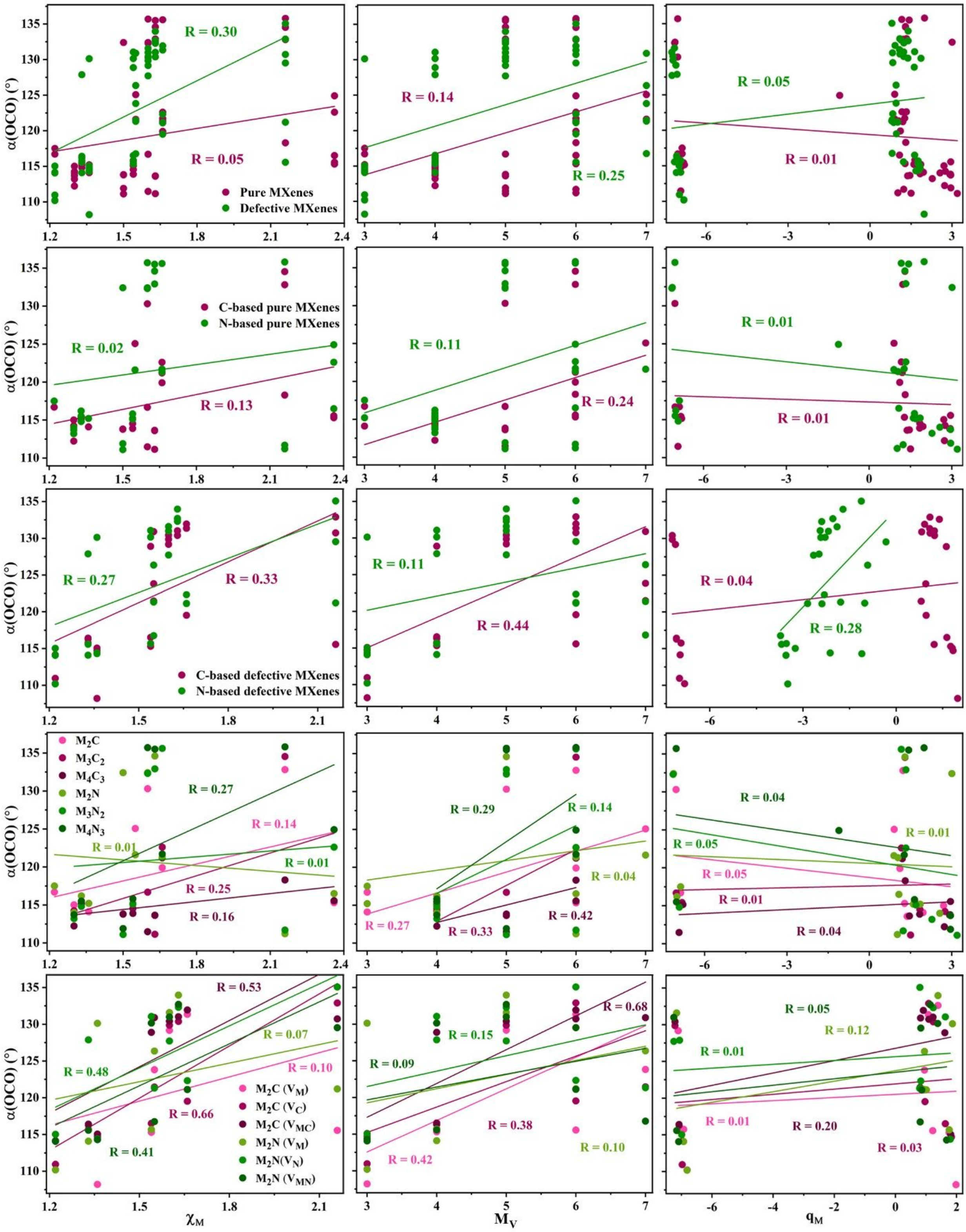
α(OCO) (°)
Pure MXenes
Defective MXenes
R = 0.30
R = 0.05
R = 0.14
R = 0.25
R = 0.05
R = 0.01
C-based pure MXenes
N-based pure MXenes
R = 0.02
R = 0.13
R = 0.11
R = 0.24
R = 0.01
R = 0.01
C-based defective MXenes
N-based defective MXenes
R = 0.27
R = 0.33
R = 0.11
R = 0.44
R = 0.04
R = 0.28
M_2C
M_3C_2
M_4C_3
M_2N
M_3N_2
M_4N_3
R = 0.27
R = 0.14
R = 0.01
R = 0.01
R = 0.25
R = 0.16
R = 0.29
R = 0.14
R = 0.04
R = 0.27
R = 0.33
R = 0.42
R = 0.04
R = 0.01
R = 0.05
R = 0.05
R = 0.01
R = 0.04
M_2C (V_M)
M_2C (V_C)
M_2C (V_{MC})
M_2N (V_M)
M_2N(V_N)
M_2N (V_{MN})
R = 0.53
R = 0.07
R = 0.10
R = 0.48
R = 0.66
R = 0.41
R = 0.68
R = 0.15
R = 0.09
R = 0.38
R = 0.42
R = 0.10
R = 0.05
R = 0.12
R = 0.01
R = 0.01
R = 0.20
R = 0.03
χ_M
M_V
q_M

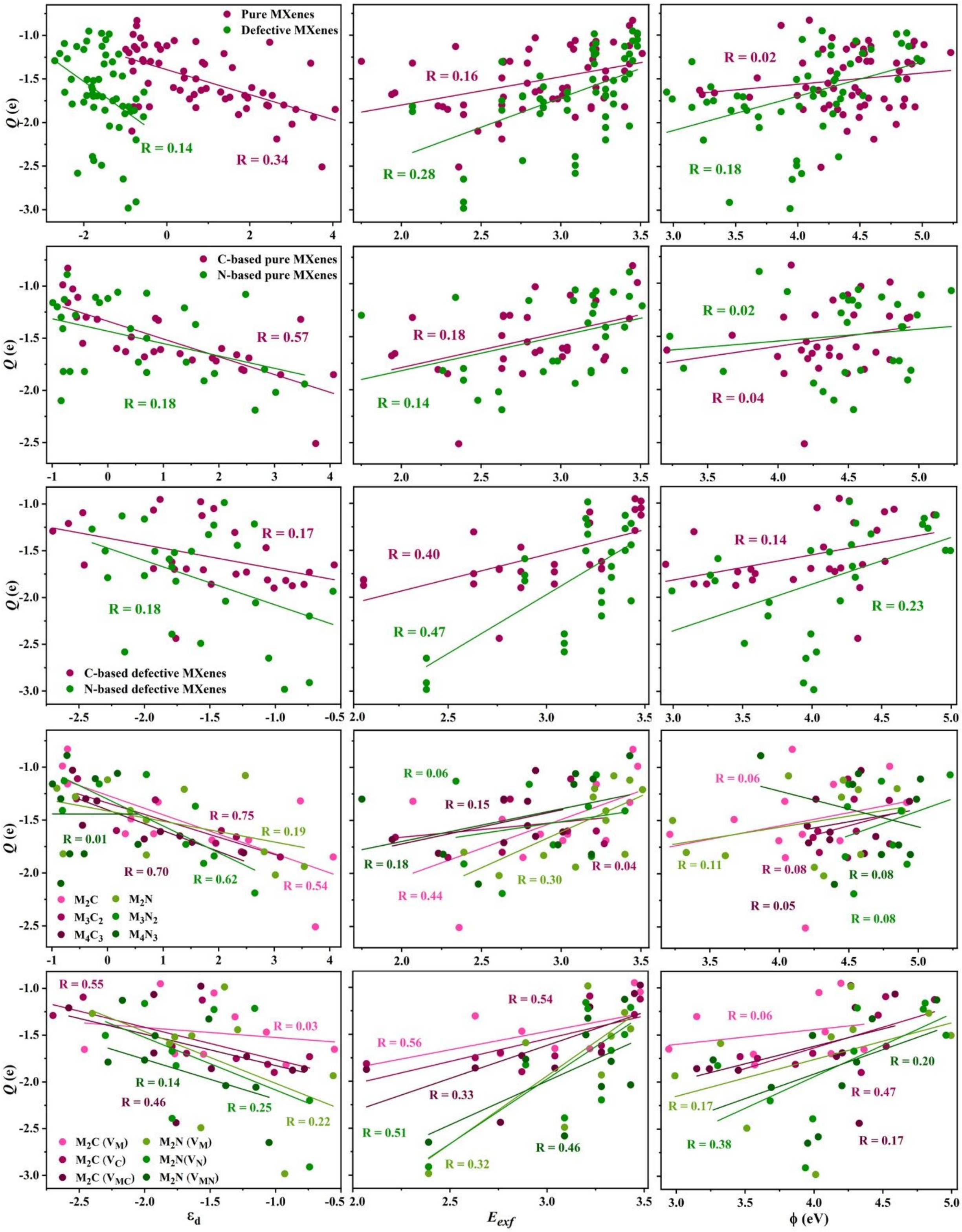

Pure MXenes
Defective MXenes
C-based pure MXenes
N-based pure MXenes
C-based defective MXenes
N-based defective MXenes
M2C
M3C2
M4C3
M2N
M3N2
M4N3
M2C (VM)
M2C (VC)
M2C (VMC)
M2N (VM)
M2N(VN)
M2N (VMN)
Q (e)
εd
Eexf
ϕ (eV)

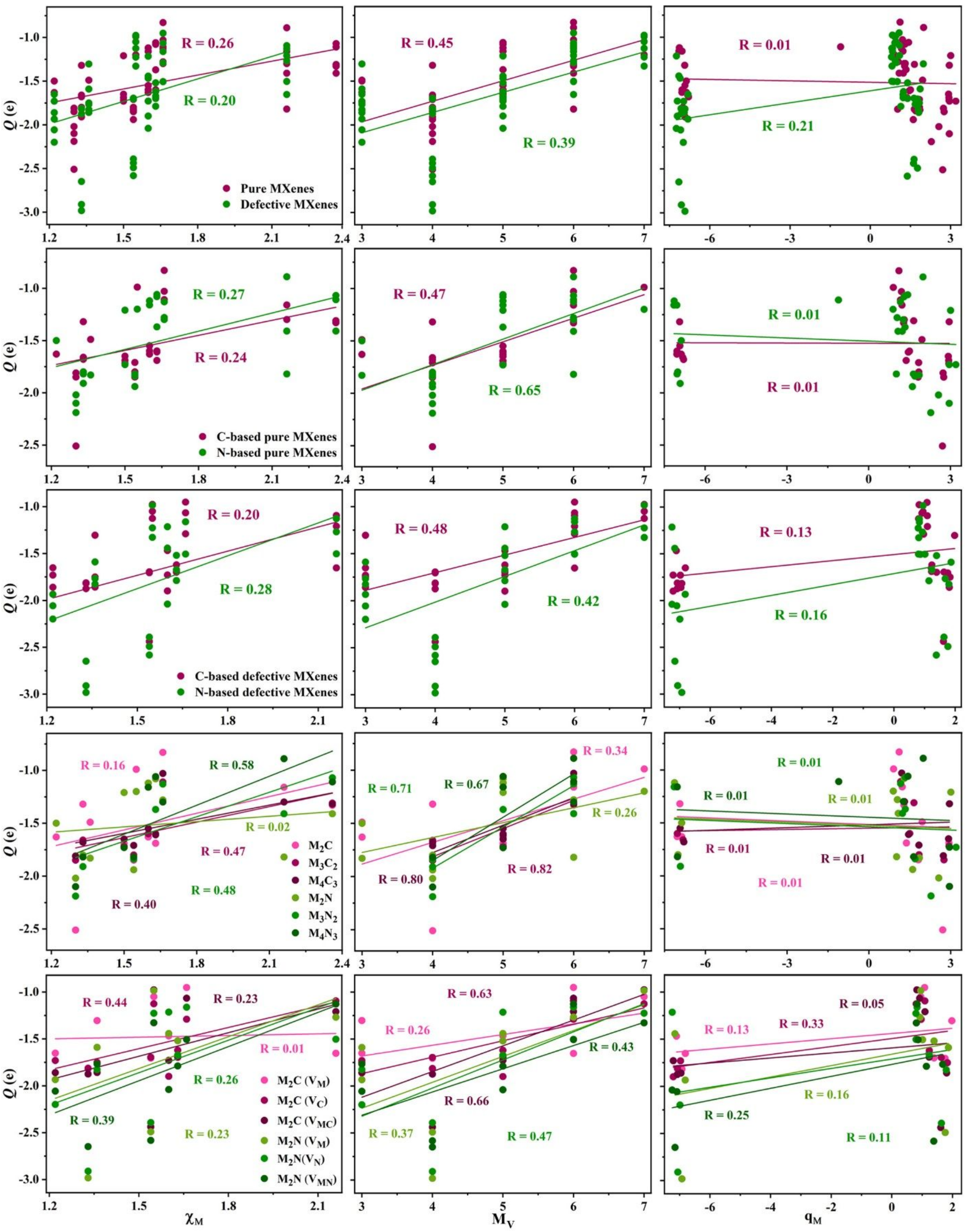

Q (e)
R = 0.26
R = 0.20
Pure MXenes
Defective MXenes
R = 0.45
R = 0.39
R = 0.01
R = 0.21
R = 0.27
R = 0.24
C-based pure MXenes
N-based pure MXenes
R = 0.47
R = 0.65
R = 0.01
R = 0.01
R = 0.20
R = 0.28
C-based defective MXenes
N-based defective MXenes
R = 0.48
R = 0.42
R = 0.13
R = 0.16
R = 0.16
R = 0.58
R = 0.02
R = 0.47
R = 0.48
R = 0.40
M2C
M3C2
M4C3
M2N
M3N2
M4N3
R = 0.34
R = 0.71
R = 0.67
R = 0.26
R = 0.80
R = 0.82
R = 0.01
R = 0.01
R = 0.01
R = 0.01
R = 0.01
R = 0.01
R = 0.44
R = 0.23
R = 0.01
R = 0.26
R = 0.39
R = 0.23
M2C (VM)
M2C (VC)
M2C (VMC)
M2N (VM)
M2N(VN)
M2N (VMN)
R = 0.63
R = 0.26
R = 0.43
R = 0.66
R = 0.37
R = 0.47
R = 0.05
R = 0.13
R = 0.33
R = 0.16
R = 0.25
R = 0.11
χM
MV
qM

S9. ML learning curves

Figure S5. MAE of the train (blue) and test set (green) *vs.* training size for the prediction of Q using HT of RFR algorithm. The shaded regions include the standard deviation arising form the cross-validation split of one hundred adjustments.

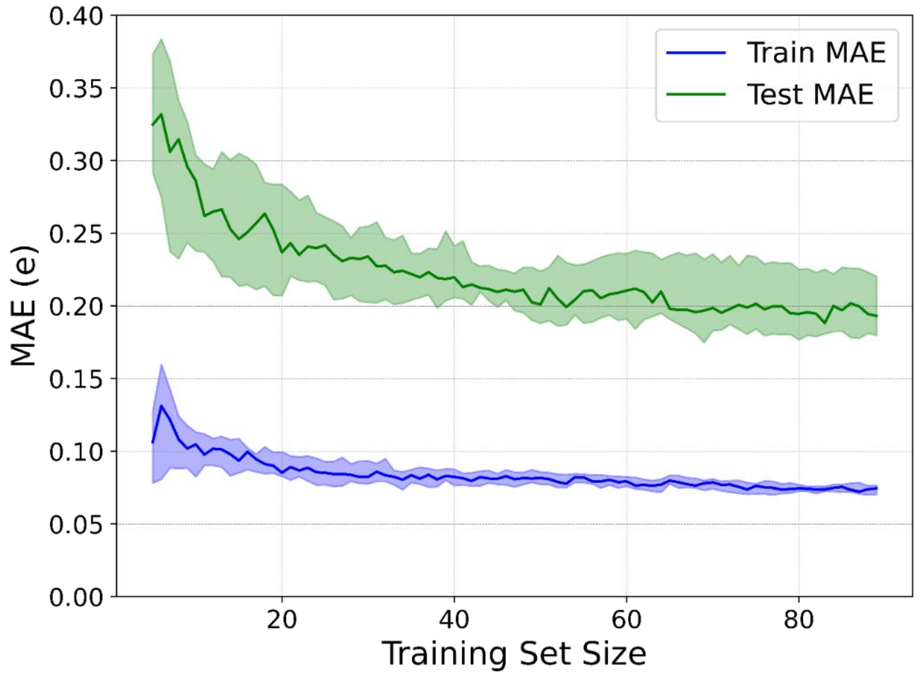

Figure S6. MAE of the train (blue) and test set (green) *vs.* training size for the prediction of *d*(CO) using HT of RFR algorithm. The shaded regions include the standard deviation arising form the cross-validation split of one hundred adjustments.

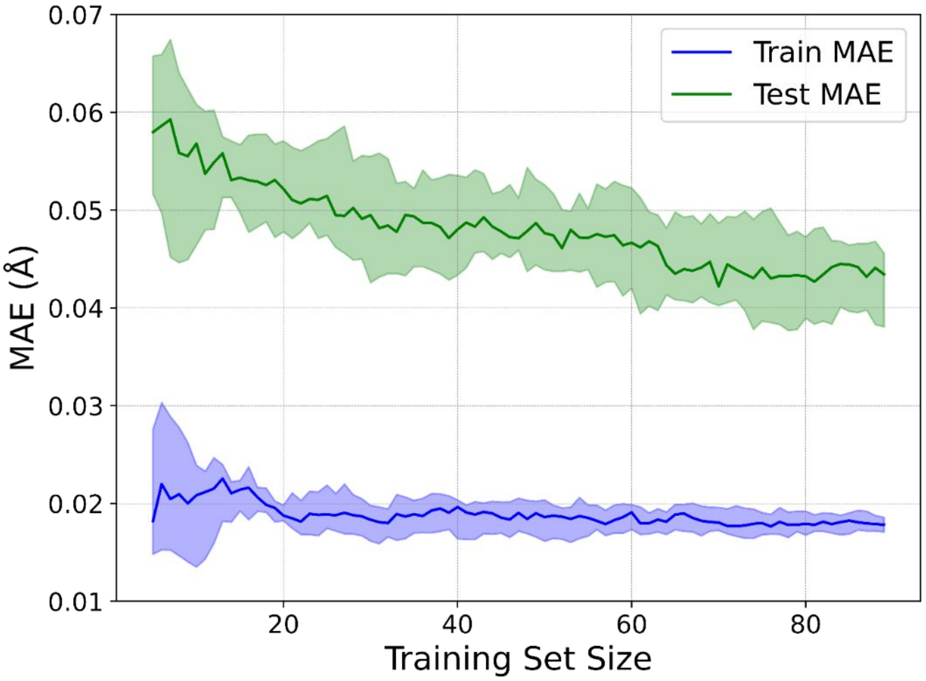

Figure S7. MAE of the train (blue) and test set (green) *vs.* training size for the prediction of α(OCO) using HT of RFR algorithm. The shaded regions include the standard deviation arising form the cross-validation split of one hundred adjustments.

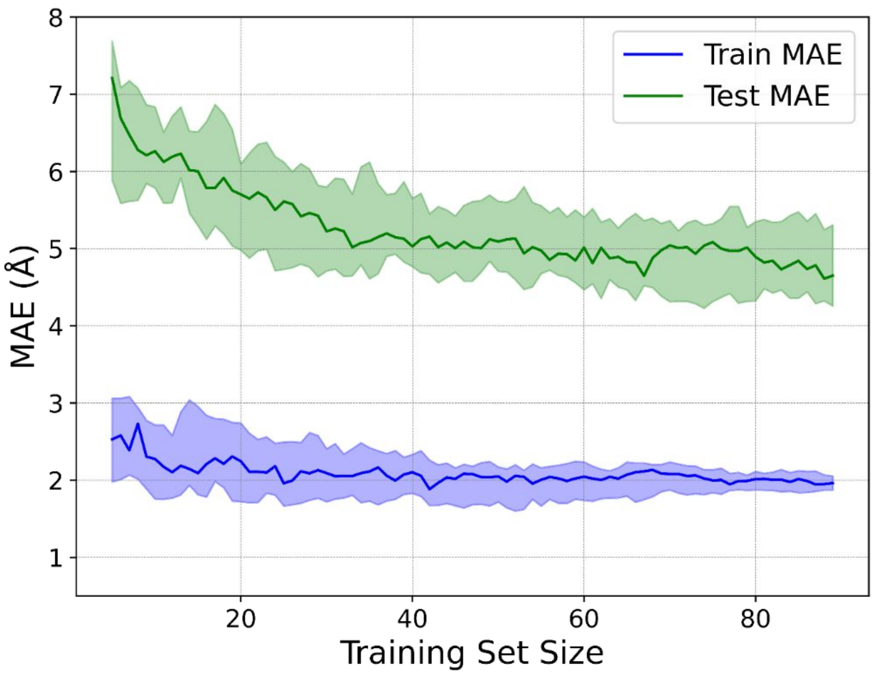

S10. Leave-one-out *vs.* recursive feature elimination

Table S1. Mean absolute error of E_{ads}, d(CO), α(OCO), and Q using leave-one-out approach and recursive feature elimination (RFE) method.

	Leave-one-out	RFE
E_{ads} /eV	0.42	0.43
Q / e	0.20	0.27
d(CO) / Å	0.04	0.04
α(OCO) / ◦	4.80	5.70

S11. Correlation between descriptors

Figure S8. Coefficient of determination heat map for the reduced set of features after leave-one-out approach for the four target properties (a) E_{ads}, (b) d(CO), (c) α(OCO), and (d) Q.

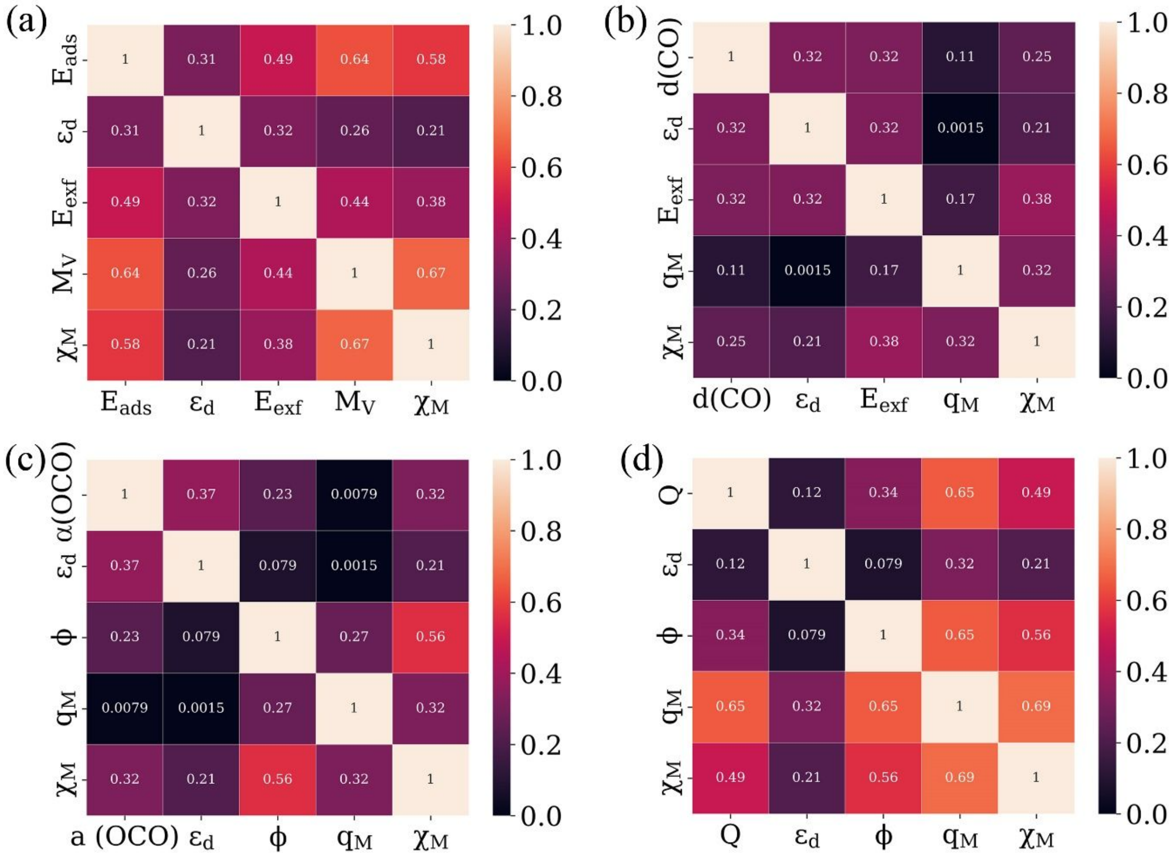

S12. References:

(1) Pedregosa, F.; Varoquaux, G.; Gramfort, A.; Michel, V.; Thirion, B.; Grisel, O.; Blondel, M.; Prettenhofer, P.; Weiss, R.; Dubourg, V.; Vanderplas, J.; Passos, A.; Cournapeau, D.; Brucher, M.; Perrot, M.; Duchesnay, E. Scikit-learn: Machine Learning in Python. *J. Mach. Learn Res.* **2011**, *12*, 2825-2830.

(2) Svetnik, V.; Liaw, A.; Tong, C.; Culberson, J. C.; Sheridan, R. P.; Feuston, B. P. Random Forest: a Classification and Regression Tool for Compound Classification and QSAR Modeling. *J. Chem. Inf. Comput. Sci.* **2003**, *43*, 1947-1958.

(3) Klusowski, J. M. Sharp Analysis of a Simple Model for Random Forests. *Arxiv* **2018** https://doi.org/10.48550/arXiv.1805.02587

(4) Genuer, R.; Poggi, J. -M.; Tuleau-Malot, C. Variable Selection using Random Forests. *Pattern Recogn. Lett.* **2010**, *31*, 2225-2236.
